# Prevalence of Hypertension in Rural Areas of China: A Meta-Analysis of Published Studies

**DOI:** 10.1371/journal.pone.0115462

**Published:** 2014-12-18

**Authors:** Xiaofang Chen, Lezhi Li, Tao Zhou, Zhanzhan Li

**Affiliations:** 1 Xiangya Nursing School, Central South University, Changsha, Hunan Province, China; 2 Nursing Department, The Third Affiliated Hostipal Of Southern Medical Universtiy, Guangzhou, Guangdong Province, China; 3 Department of Nursing, The Second Xiangya Hospital, Central South University, Changsha, Hunan Province, China; 4 Department of Cardiology, the Third Affiliated Hospital, Southern Medical University, Guangzhou, Guangdong Province, China; 5 Department of Epidemiology and Health Statistics, School of Public Health, Central South University, Changsha, Hunan Province, China; University of Oxford, United Kingdom

## Abstract

**Background:**

Hypertension is one of the leading causes of disease burden across the world. In China, the latest nationwide survey of prevalence of hypertension was ten year ago, and data in rural areas is little known. More information about hypertension prevalence could help to improve overall antihypertensive health care. We aimed to estimate the pooled prevalence of hypertension in rural areas of China.

**Methods:**

Comprehensive electronic searches of PubMed, Web of Knowledge, Chinese Web of Knowledge, Wangfang, Weipu and SinoMed databases were conducted to identify any study in each database published from January 1, 2004 to December 31, 2013, reporting the prevalence of hypertension in Chinese rural areas. Prevalence estimates were stratified by age, area, sex, publication year, and sample size. All statistical calculations were made using the Stata Version 11.0 (College Station, Texas) and Statsdirect Version 2.7.9.

**Results:**

We identified 124 studies with a total population of 3,735,534 in the present meta-analysis. Among people aged 18 years old in Chinese rural areas, the summarized prevalence is 22.81% (19.41%–26.41%). Subgroup analysis shows the following results: for male 24.46% (21.19%–27.89%, for female 22.17% (18.25%–26.35%). For 2004–2006: 18.94% (14.41%–23.94%), for 2007–2009, 21.24% (15.98%–27.01%) for 2010–2013: 26.68%, (20.79%–33.02%). For Northern region 25.76% (22.36%–29.32%), for Southern region 19.30%, (15.48%–24.08%).

**Conclusions:**

The last decade witnessed the growth in prevalence of hypertension in rural areas of China compared with the fourth national investigation, which has climbed the same level as the urban area. Guidelines for screening and treatment of hypertension in rural areas need to be given enough attention.

## Introduction

Throughout the world, cardiovascular diseases have become a major public health problem and have been recognized as a leading cause of death and disability in most developed and some developing countries [1], [2]. Hypertension is the most important risk factor for cardiovascular diseases such as stroke [3], coronary artery disease [4], [5], end-stage renal disease [6], [7] and heart failure [8]. It is estimated that about 25% of the world's adult population have hypertension, and it will be likely to increase 29% by 2025 [9]. In Europe, an estimated 37%–55% of the adult population are affected by hypertension [10], [11]. The prevalence of hypertension is even higher in some developing countries. These observations indicate the high burden of hypertension in the general population.

Since the geographic, demographic and socioeconomic characteristics are hugely different throughout China, the prevalence and awareness rates, treatments, and control of hypertension may differ widely [12]. The 1991 National Investigation Data suggested that the prevalence among Chinese adult population was 11.2%, with 16.3% in urban and 11.1% in rural areas [13]. A national epidemiology study in 2002 showed that almost 18% of Chinese people aged 15 years and older were hypertensive. The prevalence of hypertension is 19.3% in urban areas, and 18.6% in rural areas [14]. Over the decade, the prevalence of hypertension across China has increased significantly, and the rural areas had witnessed faster growth compared with the urban areas, but the gap is rapidly closing. After more than ten years since 2002, the recent meta-analysis indicated that the prevalence of hypertension was 21.5% in urban area [15], and few data are available in rural areas.

In China, about 650 million rural people are spreading in 34 regions with very diverse socioeconomic and cultural characteristics. However, nationally representative studies are scarce, and the latest nationwide survey about prevalence of hypertension was ten year ago. More relevant information can help to improve the overall antihypertensive health care. Therefore, the aims of the present study are to systematically review the studies on hypertension and to estimate the pooled prevalence of hypertension in rural areas of China.

## Materials and Methods

### Literature Search Strategy

Comprehensive electronic searches of PubMed, Web of Knowledge, Chinese Web of Knowledge (CNKI), Wangfang, Weipu (VIP) and SinoMed databases were conducted to identify any study in each database published from January 1, 2004 to December 31, 2013, reporting the prevalence of hypertension in Chinese rural areas. Related articles were identified with the following search strategy (‘hypertension’ OR ‘high blood pressure’) AND (‘prevalence’ OR ‘epidemiologic studies’) AND ‘rural’ in all databases. The relevant reference lists retrieved from databases also was searched to obtain full-scale studies. Two authors (XFC and ZZL) were entrusted to screen the titles and abstracts and reviewed the full-text of the eligible articles. When they disagree with each other, the third (LZL) makes the final decision. The search language is limited in English and Chinese. All objects included studies were approved by the Medical Ethics Committee.

### Criteria for Inclusion

In the present meta-analysis, the included studies must meet the following criteria: 1) an original epidemiological study conducted among Chinese rural population over 15 years old; 2) A cross-sectional study or data, or first phase of longitudinal study; 3) Provide information about sample size and prevalence estimation. 4) Have defined diagnostic criteria for hypertension: systolic pressure≧140 mmHg and/or diastolic pressure≧90 mmHg, or have been diagnosed with hypertension or have taken antihypertensive drug within two weeks, and secondary hypertension was excluded. 5) sample size≧300. We did not excluded these studies that were limited to a specific age group, but studies limited to a specific occupation or particular population were excluded.

### Data Extraction and Quality Assessment

Two investigators independently extracted necessary information from all included publications. Disagreements would be discussed and resolved by a third investigator if these two investigators could not reach a consensus. For all included articles, we extracted the following information: first author, publication year, screening year, province, study design, sample selection method, sample source, diagnostic criteria, method of measurement, area (northern vs southern), prevalence of overweight and obesity, onset age of study, sex ratio, case, sample, prevalence estimation, and age-specific prevalence if possible.

The quality of eligible literature was assessed according to the criteria of observational studies in recommended by Agency of Healthcare Research and Quality [16]. The tool includes 11 items: 1) Define the source of information (survey, record review); 2) List inclusion and exclusion criteria for exposed and unexposed subjects (cases and controls) or refer to previous publications; 3) Indicate time period used for identifying patients; 4) Indicate whether or not subjects were consecutive if not population-based; 5) Indicate if evaluators of subjective components of study were masked to other aspects of the status of the participants; 6) Describe any assessments undertaken for quality assurance purposes (e.g., test/retest of primary outcome measurements); 7) Explain any patient exclusions from analysis; 8) Describe how confounding was assessed and/or controlled; 9) If applicable, explain how missing data were handled in the analysis; 10) Summarize patient response rates and completeness of data collection; 11) Clarify what follow-up, if any, was expected and the percentage of patients for which incomplete data or follow-up was obtained. A maximum point of 11 were assigned to each of the items equally.

### Statistical Analysis

Because the weight of inverse variance is not optimum for fixed effects model when deal with binary data with low proportion. For the purpose of meta-analysis, the prevalence estimates were firstly transformed into a quantity (the Freeman-Tukey variant of the arcsine square root transformed proportion) suitable for usual fixed and random effects models [17]. Moreover, the transformed prevalence is weighted very slightly towards 50% and some studies with zero prevalence can still be included in the meta-analysis. The pooled proportion is calculated as the back-transform of the weighted mean of the transformed proportions, using inverse arcsine variance weights for the fixed effects model and DerSimonian-Laird weights for the random effects model [18]. Heterogeneity between studies were evaluated with Cochran chi-square (*χ^2^*) and quantified with the *I^2^* statistic, which shows the percentage of variation between studies that is due to heterogeneity rather than chance; *I^2^*<25% is considered low, 25%–50% is moderate, and >50% is treated as high-level heterogeneity [19], [20]. Considering the high heterogeneity between studies, we used random effects models for the pooled estimation of prevalence. Subgroup analysis was also conducted in order to deal with heterogeneity. Subgroups were defined as in difference in study year (2004–2006, 2007–2009, 2010–2013), sample size(<3000 vs ≧3000), area (southern vs northern), sex (male vs female), onset age of study (15–17, 18–19, 20–29, 30–39, 40–49, 50–60), age-specific group (15-, 30-, 40-, 40-, 50-, 60-, 70-), rate of overweight and obesity (20%≤, −30%, −40%,>40%.). In order to explore the potential sources of heterogeneity, meta-regression was also conducted by arranging groups of studies according to potentially relevant characteristics. Covariates examined both univariate and multivariable models were publication year, screening year, sex ratio, area, response rate, sample size (continuous), sample size (<3000 vs ≧3000), rate of overweight and obesity, quality score. Publication bias was evaluated by testing for funnel plot asymmetry, Begg's Test and Egger's Test. Significance was set at a *P* value of less than 0.05. All statistical calculations were made using the Stata Version 11.0 (College Station, Texas) and Statsdirect Version 2.7.9 (http://www.statsdirect.com).

## Results

**Figure 1:**
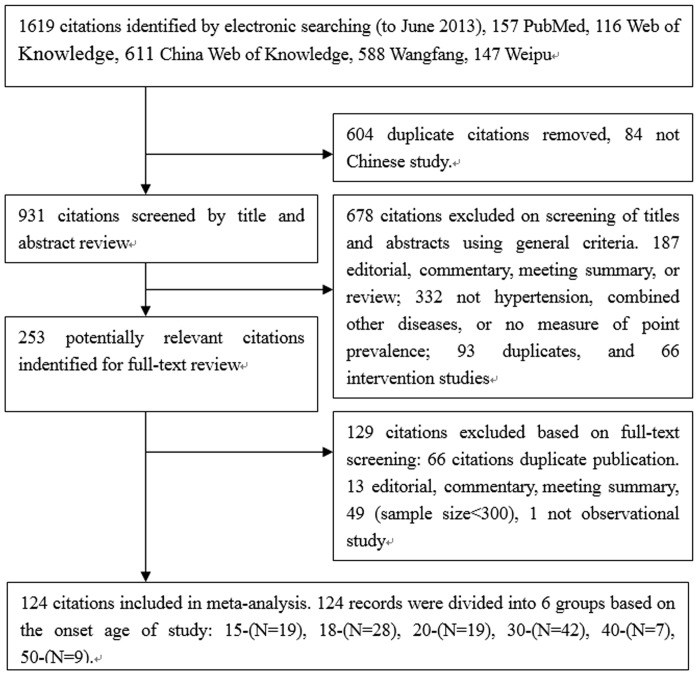
Flow diagram of included/excluded studies.

Our searches returned a total of 1619 records. After removal of duplicates and initial screening, we identified 931 records for further screening by title and abstract. After exclusion of ineligible studies, we reviewed 253 records in full. Finally, we identified 124 studies (S1 References) with a total population of 3735534 in the present meta-analysis. The 124 records were divided into 6 groups depending on the onset age of study: 15-(N = 19, n = 2551480), 18-(N = 28, n = 136249), 20-(N = 19, n = 272549), 30-(N = 42, n = 701659), 40-(N = 7, n = 48129), 50–60(N = 9, n = 25468). The flow diagram of the search process is exhibited in Fig. 1. 107 studies reported data on men (n = 1694586) and women (n = 1868799). Two studies investigated women (n = 2306) and men (n = 747) respectively. In the surveys with samples, more than 60% of the study population was women. 54 reports were from south of China (n = 431222), 70 reports were from north of China (n = 3304312), S1 Table and S2 Table shows detailed information of the 124 studies selected.

**Figure 2:**
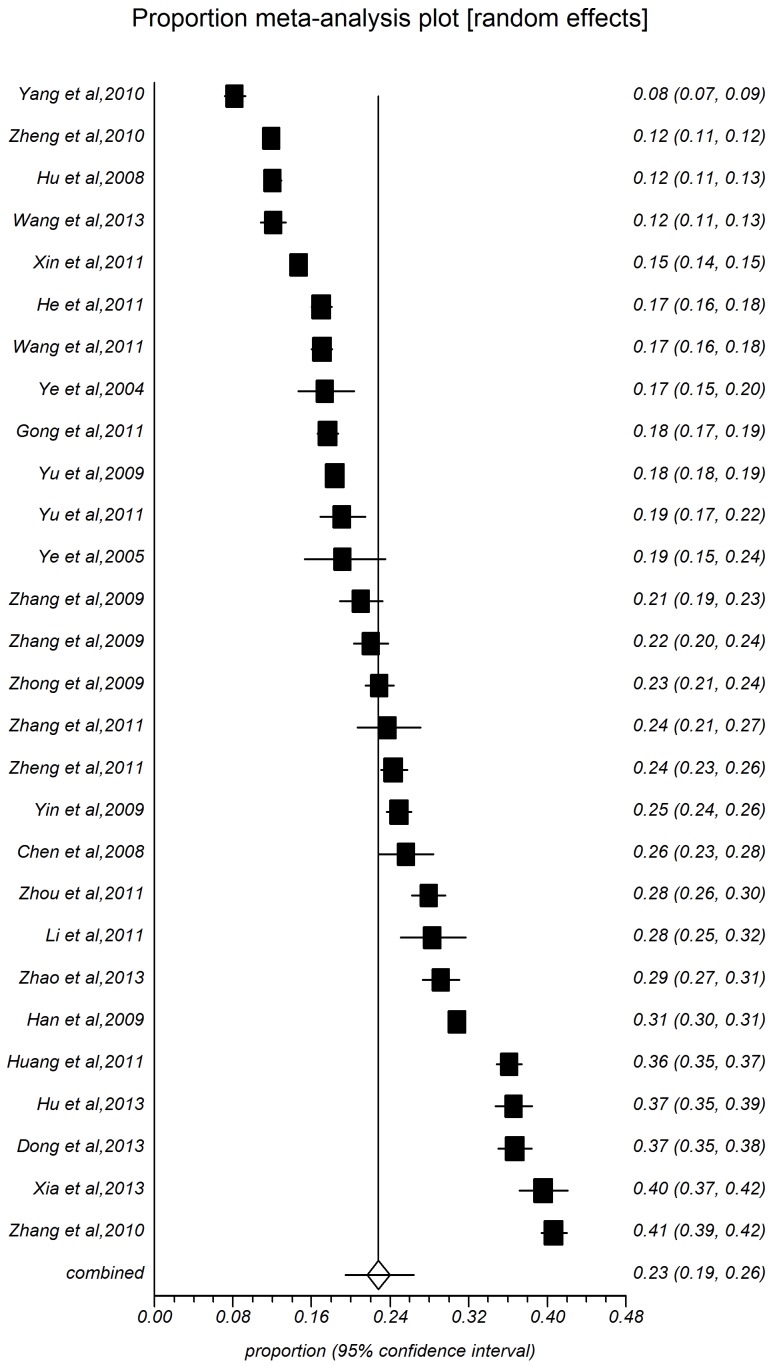
Forest plot of prevalence of hypertension for total people.

**Figure 3:**
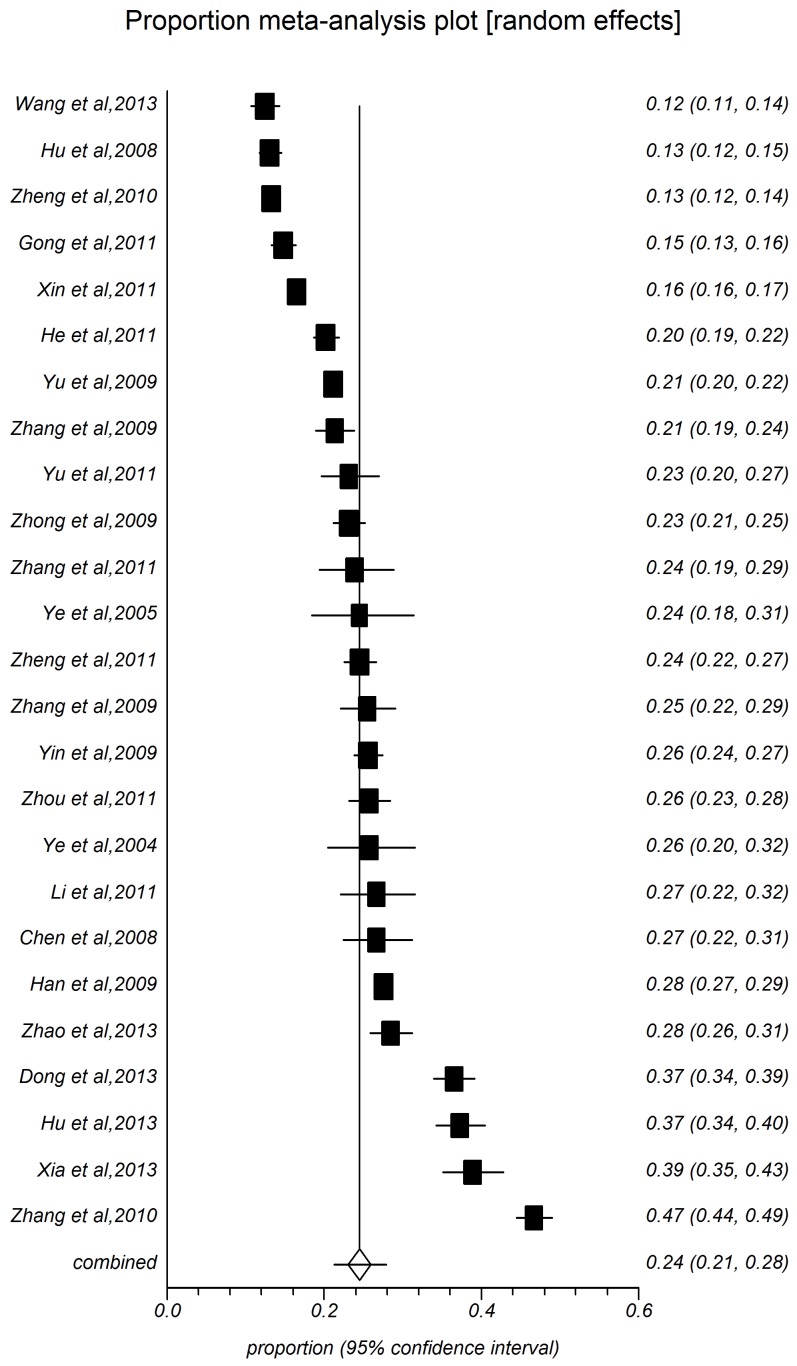
Forest plot of prevalence of hypertension for male.

**Figure 4:**
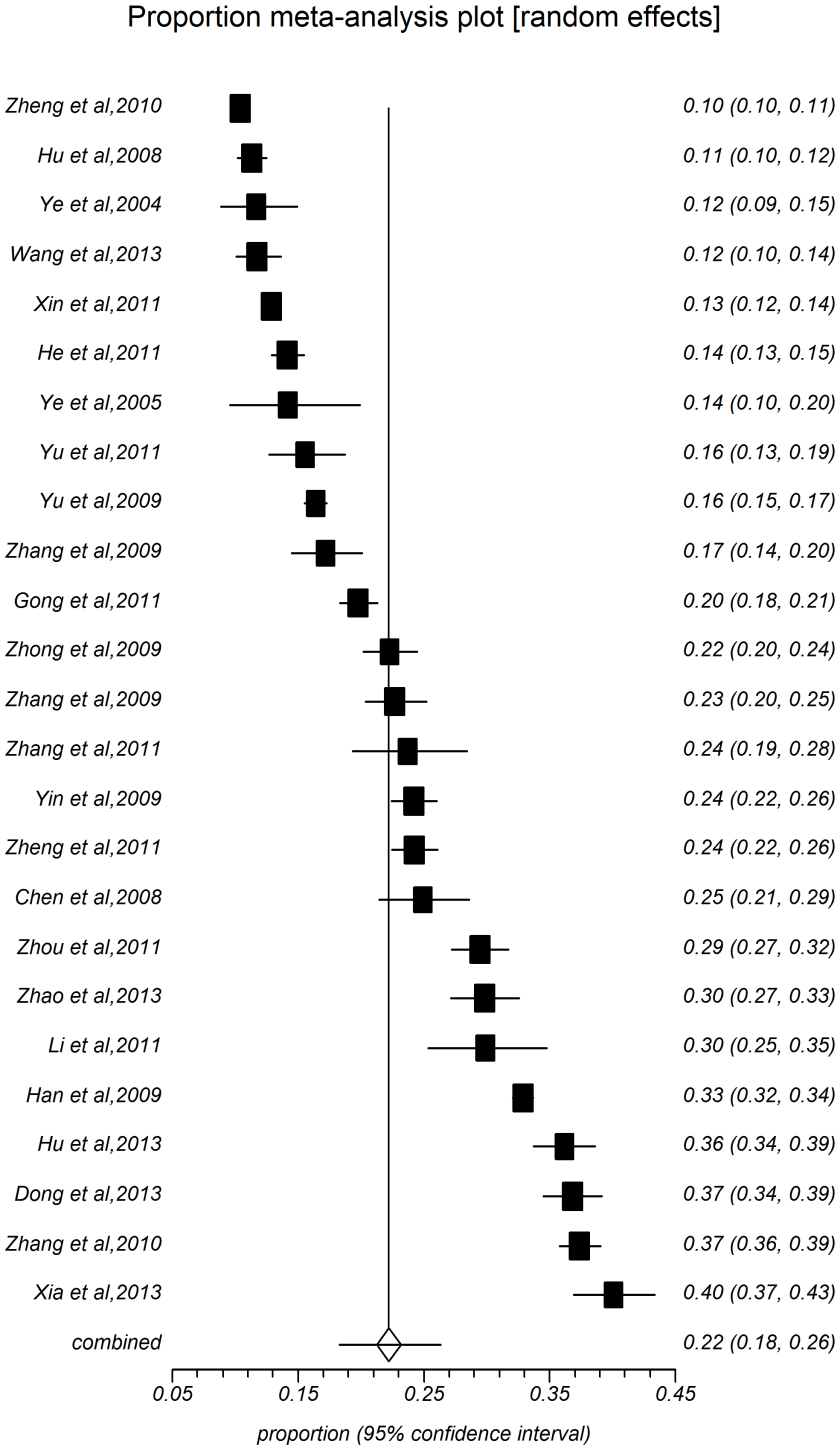
Forest plot of prevalence of hypertension for female.

The present meta-analysis focused on these studies with onset age at 18 years old [21]–[48], and other studies added to the age-specific groups. The point prevalence of hypertension with 28 study populations ranged between 8.15% and 40.67%, with an overall prevalence of 22.81% (*95%CI:* 19.41%–26.41%, Fig. 2) and high level heterogeneity between-study heterogeneity (*I^2^* = 99.6%, *P*<0.0001). Table 1 shows the pooled prevalence of all subgroups depending on study year, sample size, area, sex, age-specific group, and rate of overweight and obesity. Among people aged 18 years old in rural China, the summarized prevalence in male (24.46%, 95%CI: 21.19%–27.89%, Fig. 3) was higher than that of female (22.17%, 95%CI: 18.25%–26.35%, Fig. 4). The pooled prevalence of hypertension increased with time. The estimated prevalence was 18.94% (95%CI: 14.41%–23.94%) during 204–2006, increased to 21.24% (95%CI: 15.98%–27.01%) during 2007–2009, and the prevalence estimation was 26.68%, (95%CI: 20.79%–33.02%) during 2010–2013. The summarized prevalence of hypertension also increased with age. The estimated prevalence among age-specific groups were: for 15–30 (5.72%, 95%CI: 4.77%–6.74%), for 30-(12.08%, 95%CI: 10.65%–13.59%), for 40-(21.94%, 95%CI: 19.06%–24.97%), for 50-(33.51%, 95%CI: 30.26%–36.83%), for 60-(44.07%, 95%CI: 40.86%–47.29%), for 70-(46.02%, 95%CI: 37.40%–54.77%). The prevalence of hypertension in males increased firstly and then descended latter with age, for 15 (7.03%, 95%CI: 6.03%–8.10%), for 30 (15.06%, 95%CI: 13.22%–17.00%), for 40(23.34%, 95%CI: 20.31%–26.52%), for 50 (33.48%, 95%CI: 30.05%–37.01%), for 60 (44.90%, 95%CI: 41.52%–48.30%), and for more than 70 (45.60%, 95%CI: 37.38%–53.94%); the prevalence of hypertension for female is on the rise over age, or 15 (4.48%, 95%CI: 3.62%–6.22%), for 30 (11.04%, 95%CI: 9.26%–12.95%), for 40 (20.74%, 95%CI: 17.63%–24.03%), for 50 (32.56%, 95%CI: 29.32%–35.90%), for 60 (44.16%, 95%CI: 40.76%–47.60%), for more than 70 (49.82%, 95%CI: 38.94%–60.71%). Pooled measures of hypertension prevalence were shown for regions, different rates of overweight and obesity and sample size. Thirteen studies were included in the combined estimation for south China (I^2^ = 99.8%). The pooled prevalence was 22.81%, while the prevalence in North China (25.76%, 95%CI: 22.36%–29.32%) than that in South China (19.30%, 95%CI: 15.48%–24.08%). Prevalence increased with the growing overweight and obesity rate: rate <20% (22.43%, 95%*CI*: 17.98%–27.22%), for 30% (29.44%, 95%*CI*: 23.83%–35.38%), for 40% (30.11%, 95%*CI*: 23.51%–37.15%), and for more than 40% (30.18%, 95%CI: 24.40%–36.31%).

**Table 1:** Prevalence of hypertension according to different category.

Category	Subgroup	NO. of Studies	Prevalence (*95%CI*)(%)	*N*	*I^2^* (%)	*P*	*P*(Begg's Test)	*P*(Egger's Test)
Study year	2004–2006	6	18.94[14.41–23.94]	12783	97.7	<0.0001	0.469	0.212
	2007–2009	11	21.24[15.98–27.01]	92034	99.8	<0.0001	0.359	0.258
	2010–2013	11	26.68[20.79–33.02]	31432	99.3	<0.0001	0.445	0.095
Sample size	3000<	15	23.87[18.65–29.52]	25220	99.0	<0.0001	0.923	0.050
	≧3000	13	21.62[16.94–26.71]	111029	99.7	<0.0001	0.057	0.079
Area	Southern	13	19.30[15.48–24.08]	81947	99.8	<0.0001	0.009	0.056
	Northern	15	25.76[22.36–29.32]	54302	98.7	<0.0001	0.559	0.343
Sex	Male	25	24.46[21.19–27.89]	55664	98.8	<0.0001	0.276	0.007
	Female	25	22.17[18.25–26.35]	67795	99.4	<0.0001	0.297	0.048
Onset age of study(y)	15-	19	19.05[14.55–24.00]	2551480	99.9.	<0.0001	0.086	0.004
	18-	28	22.81[19.41–26.41]	136249	99.6	<0.0001	0.093	0.204
	20-	19	22.53[13.89–32.57	272549	100.0	<0.0001	0.100	0.007
	30-	42	32.45[30.30–34.65]	701659	99.7	<0.0001	0.897	0.869
	40-	7	31.65[19.13–40.97]	73597	99.8	<0.0001	0.562	0.019
	50–60	9	40.14[26.99–54.06]	25468	99.8	<0.0001	0.919	0.569
Age-specific group(y)	15-	34	5.72[4.77–6.74]	409203	95.9	<0.0001	0.001	0.068
	30-	56	12.08[10.65–13.59]	756328	99.3	<0.0001	0.468	0.301
	40-	50	21.94[19.06–24.97]	742333	99.8	<0.0001	0.015	0.204
	50-	49	33.51[30.26–36.83]	576708	99.7	<0.0001	0.024	0.142
	60-	52	44.07[40.86–47.29]	312863	99.5	<0.0001	0.159	0.621
	70-	34	46.02[37.40–54.77]	215334	99.9	<0.0001	0.906	0.406
Male(age)	15-	25	7.03[6.03–8.10]	216132	86.0	<0.0001	0.029	0.185
	30-	41	15.06[13.22–17.00]	378421	98.9	<0.0001	0.002	0.016
	40-	48	23.34[20.31–26.52]	354892	99.6	<0.0001	0.056	0.307
	50-	49	33.48[30.05–37.01]	273413	99.5	<0.0001	0.065	0.202
	60-	52	44.90[41.52–48.30]	157696	99.1	<0.0001	0.074	0.032
	70-	33	45.60-[37.38–53.94	102428	99.7	<0.0001	0.091	0.214
Female(age)	15-	24	4.84[3.62–6.22]	189724	96.2	<0.0001	0.423	0.503
	30-	40	11.04[9.26–12.95]	371359	99.0	<0.0001	0.116	0.253
	40-	47	20.74[17.63–24.03]	382064	99.7	<0.0001	0.102	0.092
	50-	48	32.56[29.32–35.90]	302311	99.5	<0.0001	0066	0.002
	60-	50	44.16[40.76–47.60]	156571	99.1	<0.0001	0.204	0.231
	70-	32	49.82[38.94–60.71]	112811	99.9	<0.0001	0.014	0.442
Overweight, obesity (%)	20%≤	8	22.43[17.98–27.22]	148234	99.7	<0.0001	0.904	0.473
	−30%	12	29.44[23.83–35.38]	125876	99.8	<0.0001	0.545	0.749
	−40%	11	30.11[23.51–37.15]	57081	99.7	<0.0001	0.879	0.371
	>40%	16	30.18[24.40–36.31]	62121	99.5	<0.0001	0.398	0.458

We noticed high level heterogeneity between studies and subgroups (*P*<0.001, I^2^ = 86.0%–100.0%). In univariate meta-regression analyses (Table 2), we used variables including year of publication, sample size, sex ratio (male/female), sample size (<3000 vs. ≥3000), overweight and obesity rate, and quality score to define hypertension, but did not change the estimation of prevalence. We noted that the prevalence of hypertension increased 0.39% among the participants from north China compared with the participants from the south China (meta-regression *P* = 0.046). However, in multivariable analysis, none of the factors was significantly associated with heterogeneity on meta-regression (Table 2). Egger's linear regression test (*P* = 0.093) and Begg's test (*P* = 0.204) shown no significant publication bias.

**Table 2:** Results of Meta-regression for Prevalence of Hypertension among Rural Areas of China.

Covariate	Meta-regression Coefficient	*95%* Confidence Interval	*P* value	Variance explained (%)
**Univariate analyses**				
Year of publication	0.071	−0.022 to 0.163	0.129	5.26
Year of screening	0.082	−0.006 to 0.171	0.065	9.26
Sex ratio(male vs female)	−0.533	−1.667 to 0.600	0.342	−0.23
Area(northern vs southern)	0.390	0.007 to 0.774	0.046	11.27
Response rate	−6.694	−15.164 to 1.776	0.116	5.97
Sample size, continuous	−0.00002	−0.00005 to 0.0002	0.373	−0.61
Sample size(<3000 vs ≧3000)	−0.023	−0.436 to 0.390	0.909	−3.81
Overweight, obesity (%)	0.081	−0.003 to 0.040	0.089	11.95
Quality score	−0.069	−0.254 to 0.116	0.451	−1.52
**Multivariable analyses**				−0.99
Year of publication	0.097	−0.421 to 0.615	0.672	
Year of screening	−0.002	−0.556 to 0.552	0.994	
Sex ratio(male vs female)	−1.079	−4.433 to 2.275	0.472	
Area(northern vs southern)	0.444	−0.388 to 1.278	0.248	
Response rate	−10.182	−35.162 to 15.098	0.373	
Sample size, continuous	−0.00002	−0001 to 0.00007	0.530	
Sample size(<3000 vs ≧3000)	0.453	−0.580 to 1.488	0.334	
Overweight, obesity (%)	−0.006	−0.051 to 0.038	0.740	
Quality score	−0.161	−525.55 to 164.873	0.257	

## Discussion

There are no nationwide data regarding the prevalence of hypertension in rural areas of China. This is the first report attempting to synthesize the prevalence estimations of hypertension among Chinese rural population by using meta-analysis. This systematic review with meta-analysis of observational studies done in China included 124 reports done in China in the last decade. Therefore, it is possible to provide a reliable estimate of prevalence. A national epidemiology study in 2002 found that almost 18% of Chinese aged 15 years and older were hypertensive [14]. This meta-analysis indicates that the prevalence of hypertension in rural areas of China is 22.8% (≥18 years old) in the last decade, which is well-above the level in 2002 (18.0%) of national investigation and even 1.3% higher than the prevalence (21.5%) of urban. The present level of Chinese rural areas has reached an epidemic proportion, and prevalence is still increasing on the time. Several reasons account for the current situation. For such a widely prevalent disease, even modest improvement in interventions can profoundly impact the population level. Therefore, prevention measures aimed at high-risk populations should be taken immediately.

Our prevalence estimation of hypertension for rural residents was not different from previous reports in other countries. In India, the prevalence of hypertension was 19.0% in a rural community [49]. In 2008, a cross-sectional study of rural community health status among 1078 adults (aged >or  = 18 years) shown that the crude prevalence of hypertension was 18.3% in Nigeria while a total of 30 peer-reviewed publications were identified that reported the prevalence of hypertension in 33 143 patients was 32.6% from rural Ibero-America [50]. A study suggested that the prevalence of hypertension in young men is higher than in young women [51], but the situation was reverse before the age of 40, and after that the prevalence of hypertension did not differ between males and females in rural areas. The prevalence of hypertension were substantially different in regions. The prevalence was higher in north China than in south China (25.7% vs 19.3%), and difference still exists between urban and rural population [14] (25.8% vs 20.4%), which may be attributed to discrepancy in eating habits. The proportion of salt intake among the local residents in Hainan and the long-term living from the north is 7.2% and 8.4%, while the short-term residents from the north is more than16.9% [52]. Such findings should be confirmed by investigation with a larger sample size. Overweight and obesity are also important risk factors of hypertension. The pooled results indicated that the prevalence increases with the growing rate of overweight and obesity. The proportion of overweight and obesity among Chinese population is 38.5% according to the latest data [53].

However, a challenge prevailing in Chinese rural areas is the lower levels of awareness rate, treatment and control of hypertension. The hypertension awareness rates in rural and urban areas were 13.5% vs. 35.6% in 1999, 34.2% vs. 51.2% in 1998, and 22.5% vs. 41.1% in 2002. Despite great improvements, the hypertension awareness rate in rural areas is still significantly lower than the urban areas [54]. Meanwhile, treatment rates of hypertension in urban and rural areas were 35.6% vs. 13.9% in 1999, 41.4% vs. 21.4% in 1998 [55], [56], and 35.1% vs. 17.4% in 2002. Over the ten years, the hypertension treatment rate in rural areas was rising, but not significant. There is still an obvious gap between China and the developed countries. For instance, the United States was 59.0% in 2000 [57]. The rate of blood pressure control is at very low level. The rates in urban and rural areas were 4.1% vs. 2.8% in 1991 [54], 4.4% vs. 2.6% in 1998 [55], and 9.7% vs. 3.5% in 2002 [56]. The disparities in hypertension prevalence and control among population indicate a need for interventions that span the population and focus on vulnerable groups.

Although this meta-analysis includes more studies encompassing larger sample sizes than individual studies, some limitations need to be illustrated clearly. First, the whole analysis shows higher heterogeneity, but most of the studies had large sample sizes that can produce very precise estimation. In addition, meta-regression analysis did not show that any factors that may be associated with the prevalence of hypertension. We still considered that other factors may influence heterogeneity and partly contribute to the increased prevalence of hypertension, such as dietary fats, high intake of sodium, stressful life, sedentary lifestyle urbanization and industrialization. Unfortunately, we did not obtain enough information about these aspects for further analysis. Besides, specific statistical comparisons with the previous results in all subgroups have not been done because of the data limitation, different grouping methods and unspecific analysis from the previous. Considering the huge rural population base, a little bit of growth in prevalence may have important implication for public health. Second, the included studies the meta-analysis are almost cross-sectional design. We did not identify any longitudinal studies, which may be difficult to carry out but quite important for further research because it could provide information on the development of hypertension. Third, although the same standard diagnosis of hypertension was adopted, the time point and time of blood pressure measurement may have an influence on the identification of hypertension. Another problem is the impossibility of identifying white-coat hypertension, which affects the pooled results because it occurs in 15% to 30% of subjects with an elevated office blood pressure [58], [59]. Finally, there are some disparities in the distribution of healthcare resources in China. Some low-income residents did not have resources for examination, and we have restricted sample size in inclusion criteria. The potential information bias may have an effect on the pooled rates.

In conclusion, the last decade witnessed the growth in prevalence of hypertension in rural areas of China compared with the fourth national investigation, which has climbed the same level as the urban area. Guidelines for screening and treatment of hypertension in rural are need to be given enough attention.

## Supporting Information

S1 Table **Characteristic of Studies on the Prevalence of hypertension.**
(DOC)

S2 Table **Characteristic of Studies on the Prevalence of hypertension (continued Table S1).**
(DOC)

S1 Checklist **PRISMA Checklist.**
(DOC)

S1 Diagram **PRISMA Flow Diagram.**
(DOC)

S1 References **References included in the meta-analysis.**
(DOC)

## References

[1] Lopez AD, Mathers CD, Ezzati M, Jamison DT, Murray CJ (2006) Global and regional burden of disease and risk factors, 2001: systematic analysis of population health data. Lancet 367:1747–1757. 16731270 10.1016/S0140-6736(06)68770-9

[2] He J, Gu D, Wu X, Reynolds K, Duan X, Y, et al. (2005) Major causes of death among men and women in China. N Engl J Med 353:1124–1134. 16162883 10.1056/NEJMsa050467

[3] Kannel WB, Wolf PA, Verter J, McNamara PM (1996) Epidemiologic assessment of the role of blood pressure in stroke: the Framingham Study. 1970 [see comments]. JAMA 276:1269–1278. 8849757

[4] van der Hoogen PC, Feskens EJ, Nagelkerke NJ, Menotti A, Nissinen A, et al (2000) The relation between blood pressure and mortality due to coronary heart disease among men in different parts of the world. Seven Countries Study Research Group. N. Engl. J. Med 342:1–8. 10620642 10.1056/NEJM200001063420101

[5] Flack JM, Neaton J, Grimm RJ, Joanna S, Jeffrey C, et al (1995) Blood pressure and mortality among men with prior myocardial infarction. Multiple Risk Factor Intervention Trial Research Group. Circulation 92:2437–2445. 7586343 10.1161/01.cir.92.9.2437

[6] Klag MJ, Whelton PK, Randall BL, Neaton JD, Brancati FL, et al (1996) Blood pressure and end-stage renal disease in men. N Engl J Med 334:13–18. 7494564 10.1056/NEJM199601043340103

[7] Tozawa M, Iseki K, Iseki C, Kinjo K, Ikemiya Y, et al (2003) Blood pressure predicts risk of developing end-stage renal disease in men and women. Hypertension 41:1341–1345. 12707291 10.1161/01.HYP.0000069699.92349.8C

[8] Levy D, Larson MG, Vasan RS, Kannel WB, Ho KKL (1996) The progression from hypertension to congestive heart failure. JAMA 275:1557–1562. 8622246

[9] Mittal BV, Singh AK (2010) Hypertension in the developing world: challenges and opportunities. Am J Kidney Dis 55:590–598. 19962803 10.1053/j.ajkd.2009.06.044

[10] Wolf-Maier K, Cooper RS, Banegas JR, Giampaoli S, Hense HW, et al (2003) Hypertension prevalence and blood pressure levels in 6 European countries, Canada, and the United States. JAMA 289:2363–2369. 12746359 10.1001/jama.289.18.2363

[11] Firmann M, Mayor V, Vidal PM, Bochud M, Pecoud A, et al (2008) The CoLaus study: a population-based study to investigate the epidemiology and genetic determinants of cardiovascular risk factors and metabolic syndrome. BMC Cardiovasc Disord 8:6. 18366642 10.1186/1471-2261-8-6 PMC2311269

[12] Reynolds K, Gu D, Muntner P, Wu X, Chen J, et al (2003) InterASIA Collaborative Group. Geographic variations in the prevalence, awareness, treatment and control of hypertension in China. J Hypertens 21:1273–1281. 12817173 10.1097/00004872-200307000-00014

[13] PRC National Blood Pressure Survey Cooperative Group (1995) A summary on the 1991 national sampled study on hypertension in China. Chin J Hypertens 3:1–2.

[14] Department of Disease Control and Prevention, Ministry of Health (2006) Report on chronic diseases in China. Beijing, China: Chinese Centre for Disease Control and Prevention.

[15] Ma YQ, Mei WH, Yin P, Yang XH, Rastegar SK, et al (2013) Prevalence of hypertension in Chinese cities: a meta-analysis of published studies. PLoS One 8:e58302. 23484011 10.1371/journal.pone.0058302 PMC3590128

[16] Rostom A, Dubé C, Cranney A, Saloojee N, Richmond Sy, et al. (2004) Celiac Disease. Rockville (MD): Agency for Healthcare Research and Quality (US); (Evidence Reports/Technology Assessments, No. 104.) Appendix D. Quality Assessment Forms. Available: http://www.ncbi.nlm.nih.Gov/books/NBK35156/ Accessed 2014 March 5.

[17] Stuart A, Ord JK (1996) Kendall's Advanced Theory of Statistics. 6th Ed. London: Edward Arnold.

[18] DerSimonian R, Laird N (1986) Meta-analysis in Clinical Trials. Controlled Clinical Trials 7:177–188. 3802833 10.1016/0197-2456(86)90046-2

[19] Higgins J, Thompson SG (2002) Quantifying heterogeneity in a meta-analysis. Stat Med 21:1539–58. 12111919 10.1002/sim.1186

[20] Higgins J, Thompson SG, Deeks JJ, Altman DG (2003) Measuring inconsistency in meta-analyses. BMJ 327:557. 12958120 10.1136/bmj.327.7414.557 PMC192859

[21] Zhang AH, Zhang SW (2009) Investigation on the prevalence of hypertension in rural residents aged over 18 in Zoucheng Ctiy in 2006. Prev Med Trib 15(8):720–721.

[22] Yu YX, Yang XC (2009) Investigation of check-up among rural residents in 2008. Med J Chin People Health 21:2364–2366.

[23] Zhou YX (2011) Survey on hypertension among rural residents in Huangcheng Township, Weishan County. Pre Med Trib 17:909–913.

[24] Yu QH (2011) Investigation on the prevalence of chronic non-communicable disease in rural residents aged over 18 in Yutai County. World Health Dig Med. Periodieal 8:13–14.

[25] He F, Qiu M, Chen WG, Xu JJ, Zhu HB, et al (2012) Major chronic diseases and risk factors survey of rural residents in Yandu District, 2011. Chin J dis Control Prev 16:560–563.

[26] Zhong DC, Cao WZ, Zhang T, Yan H, Xiong Z (2009) Analysis of the prevalence of hypertension and its risk factors among rural population in Zigong City. Sichuan Med J 30:1056–1058.

[27] Xia YY, Li G, Ding XB, Mao DQ, QI L, et al (2013) Prevalence of hypertension and its associated factors among rural residents in Chongqing. J Trop Med 13:350–353.

[28] Zheng ZH, Liang PS, Kuang ZP (2010) Study on prevalence and related factors of chronic diseases among adults in rural area of Zhongshan city. China Chlin Prac Med 4:219–220.

[29] Wang KW, Cai L, Shu ZK, Dong J, Ye YH, et al (2011) Relationship between prevalence of cardiovascular diseases and clustering of risk factors among rural residents in Luoping county of Yunnan province. Chin J Public Health 27:1291–1292.

[30] Ye HF, Liu XM, Su YA, Zhao XB, Luo BH (2005) Analysis of prevalence features of hypertension and influential factors in rural inhabitants in Yingfe City. Chin Trop Med 5:1114–1116.

[31] Dong CX, Ge PF, Ren XL, Fan HQ, Yan X (2013) Prevalence, awareness, treatment and control of hypertension among adults in rural north-western China: a cross-sectional population survey. J Internal Med Res 41:1291–1300. 10.1177/0300060513488498 23816929

[32] Zhang G, Zhu CY, Zhang ZF, Duan JJ, Xia J, et al (2009) Investigation on hypertension in rural adults in Wuhan. 20:24–26.

[33] Li Y (2011) The Population distribution of hypertension and risk factors in the countryside of Shenyang. Journal of Shenyang Medical College 13:228–230.

[34] Zheng YT, Wang Y (2011) Analysis of prevalence of hypertension and its risk factors in rural areas of Shenyang city. Chin Med J Metall Indus 28:573–574.

[35] Yin CG, Ma YX, Wang SL, Xu TL, Yan P (2009) Epidemiology study of hypertension in rural areas of Shandong province. Prev treatment of Cardio-cerebral-vascular Dis 9:137–138.

[36] Yang HJ, Zhou ZH, Wang JJ (2010) Analysis of influencing factors of hypertension in rural community. Chin J General Prac 8:896–898.

[37] Zhang Y, Liu WX, Shi ZY, Li JY, Gao YZ (2011) Hypertension prevalence and related factors among residents in rural areas of Ji County. Ningxia Med J 33:1222–1223.

[38] Gong J, Jia SB, Ma L (2011) Prevalence of hypertension and its risk factors among rural residents in Ningxia. Chin Prev Med 12:227–229.

[39] Hu XJ, Chen H, Dong YP, Chu XC, Lian YS (2013) Epidemiological survey of hypertension among rural resident in Wujiang District of Jiangsu province. Chin Health Service Management 5:391–393.

[40] Chen B, Liu JY, Sun L, Wang JF, Yang JX, et al (2008) Prevalence and risk factors of hypertension in rural communities in Henan. Moedern Prev. Med 35:2823–2826.

[41] Han B, Yu DH, Wang CJ, Ping GZ, Lu J, et al (2009) Prevalence, awareness, treatment and control of hypertension in rural areas of Henan. J Zhengzhou Univ 44:337–339.

[42] Xin RB, Meng GY, He L, Lu J, Huang WY, et al (2011) Sampling survey of hypertension in Zhuang nationality in Tiandong County. Guangxi Med 33:963–965.

[43] Zhao JQ (2013) Investigation on current situation of hypertension and its influencing factors in rural population in Xihe county of Gansu province. Bull Dis Control Prev 28:27–29.

[44] Ye ZW, Xiang QY (2011) Surveillance report on nutritional and health conditions of the rural residents in Juchao District, Chaohu city. Anhui J Prev Med 17:190–191.

[45] Hu HH (2008) Survey on the prevalence, Knowledge, treatment and control in rural area of Changshan County. Prev and Treatment Cardio-Cerabral Vascular Dis 8:195–196.

[46] Zhang LJ, He SP, Liu YF, Liu CX (2010) Analysis on results of physical examination among rural residents Huairou district of Beijing city. Occup and Health 26:1872–1874.

[47] Wang YM. Zhu JM, Li BK, Hu GH (2013) Investigation of epidemic characters of hypertension disease of rural residents in Anhui province. J Benbu Med Coll 38:321–323.

[48] Huang JP, Zhang W, Li XH, Zhou JX, Gao Y, et al (2001) Analysis of the prevalence and risk factors oh hypertension in the She population in Fujian, China. Kidney Blood Press Res 34:69–74. 10.1159/000323164 21212687

[49] Kokiwar PR, Gupta SS, Durge PM (2012) Prevalence of hypertension in a rural community of central India. J Assoc Physicians India 60:26–29. 23409417

[50] Diaz AA, Tringler MF (2014) Prevalence of hypertension in rural populations from Ibero-America and the Caribbean. Rural Remote Health 14:2591. 24484198

[51] Wu X, Duan X, Gu D, Hao J, Tao S, et al (1995) Prevalence of hypertension and its trends in Chinese populations. International Journal of Cardiology 52:39–44. 8707434 10.1016/0167-5273(95)02443-z

[52] Lin K, Lin L, He CM, Pang MW, Chen HL (2014) Epidemic characteristics of hypertension in South and North China. Med J Wuhan Univ 35:114–117.

[53] Ni GH, Zhang J, Zheng FT (2013) Status and trends of Chinese obesity epidemic. Food Nutri Chin 19:70–74.

[54] Li LM (2008) Chinese residents of nutrition and health survey. Beijing: People's Medical Publishing House.

[55] Ministry of Health of the People's Republic of China (2005) China's prevention and control of hypertension guidelines.

[56] Hu TH, Li LM, Cao WH, Zhan SY, Li F, et al (2000) Community-based comprehensive prevention and control of hypertension in China (CCPACH Study)-prevalence and epidemiological characteristics in urban and rural area. Chin J Epidemiology 21:177–180. 11860779

[57] Chobanian AV, Bakris GL, Black HR, Cushman WC, Green LA, et al (2003) The Seventh Report of the Joint National Committee on Prevention, Detection, Evaluation, and Treatment of High Blood Pressure: the JNC 7 report. JAMA 289:2560–2572. 12748199 10.1001/jama.289.19.2560

[58] O'Brien E, Parati G, Stergiou G, Asmar R, Beilin L, et al (2013) European society of hypertension position paper on ambulatory blood pressure monitoring. J Hypertens 31:1731–1768. 24029863 10.1097/HJH.0b013e328363e964

[59] O′Brien E, Coats A, Owens P, Petrie J, Padfield PL, et al (2000) Use and interpretation of ambulatory blood pressure monitoring: recommendations of the British hypertension society. BMJ 320:1128–1134. 10775227 10.1136/bmj.320.7242.1128 PMC1127256

