# Supplementary material for: Prevalence of Hypertension in Rural Areas of China: A Meta-Analysis of Published Studies
Source: PLoS One. 2014 Dec 18;9(12):e115462. doi: 10.1371/journal.pone.0115462 (PMC4270770; doi:10.1371/journal.pone.0115462)
Supplement: S1 Table — Characteristic of Studies on the Prevalence of hypertension. (DOC) [file pone.0115462.s001.doc]

**Supplementary 2 Characteristic of Studies on the Prevalence of hypertension**

| **NO.** | **First author** | **Publication**  **Year** | **Screening**  **Year** | **Province** | **Study design** | **Area** | **Overweight and**  **Obesity (%)** | **Minimum age** | **Sex**  **(M/F)** | **Case**  **(n)** | **Sample**  **size** | **Prevalence (%)** |
| --- | --- | --- | --- | --- | --- | --- | --- | --- | --- | --- | --- | --- |
| 1 | Yuan et al. | 2009 | 2006 | Shanxi | Cross-sectional | Northern | … | 25 | 0.95 | 301 | 1256 | 0.24 |
| 2 | Lin et al. | 2008 | 2006 | Zhejiang | Cross-sectional | Southern | … | 60 | 1.07 | 241 | 1853 | 0.13 |
| 3 | Zhang et al. | 2008 | 2004 | Jiangsu | Cross-sectional | Southern | 39.2 | 20 | 0.74 | 2671 | 4976 | 0.54 |
| 4 | Jie et al. | 2007 | 2004 | Tianjin | Cross-sectional | Northern | … | 15 | 0.92 | 754783 | 2390754 | 0.32 |
| 5 | Zhang et al. | 2008 | 2006 | Sichuan | Cross-sectional | Northern | … | 25 | 1.09 | 3377 | 137674 | 0.02 |
| 6 | Lin et al. | 2009 | 2006 | Shandong | Cross-sectional | Northern | 23.7 | 20 | 0.71 | 18801 | 57197 | 0.33 |
| 7 | Zhang et al. | 2009 | 2006 | Shandong | Cross-sectional | Northern | 35.0 | 18 | 0.86 | 282 | 1344 | 0.21 |
| 8 | Yu et al. | 2009 | 2008 | Guangdong | Cross-sectional | Southern | 21.4 | 18 | 0.72 | 2130 | 11607 | 0.18 |
| 9 | Sun et al. | 2008 | 2005 | Neimenggu | Cross-sectional | Northern | … | 35 | 0.98 | 17147 | 45390 | 0.38 |
| 10 | Liu et al. | 2011 | 2010 | Hunan | Cross-sectional | Southern | 30.6 | 20 | … | 532 | 1989 | 0.27 |
| 11 | Zhou et al. | 2011 | 2010 | Shandong | Cross-sectional | Northern | 36.2 | 18 | 0.69 | 748 | 2681 | 0.28 |
| 12 | Yu et al. | 2011 | 2010 | Shandong | Cross-sectional | Northern | 30.4 | 18 | 0.89 | 212 | 1110 | 0.19 |
| 13 | He et al. | 2011 | 2011 | Jiangsu | Cross-sectional | Southern | … | 18 | 0.89 | 857 | 5051 | 0.17 |
| 14 | Zhong et al. | 2009 | 2005 | Sichuan | Cross-sectional | Southern | … | 18 | 1.16 | 716 | 3125 | 0.23 |
| 15 | Yu et al. | 2010 | 2008 | Shandong | Cross-sectional | Northern | 37.7 | 35 | 0.77 | 3232 | 8951 | 0.36 |
| 16 | Li et al. | 2013 | 2010 | Hebei | Cross-sectional | Northern | … | 40 | 0.78 | 445 | 1040 | 0.43 |
| 17 | Zhou et al. | 2011 | 2011 | Guangdong | Cross-sectional | Southern | … | 15 | 0.87 | 154 | 641 | 0.24 |
| 18 | Xia et al. | 2013 | 2013 | Chongqing | Cross-sectional | Southern | 45.7 | 18 | 0.70 | 605 | 1528 | 0.40 |
| 19 | Zheng et al. | 2010 | 2007 | Guangdong | Cross-sectional | Southern | 30.8 | 18 | 0.94 | 1850 | 15550 | 0.12 |
| 20 | Su et al. | 2013 | 2013 | Zhejiang | Cross-sectional | Southern | … | 35 | 6.20 | 36560 | 125479 | 0.29 |
| 21 | Fan et al. | 2013 | 2008 | Zhejiang | Cross-sectional | Southern | … | 15 | … | 3728 | 38378 | 0.10 |
| 22 | Tao et al. | 2009 | 2007 | Yunnan | Cross-sectional | Southern | … | 35 | 0.65 | 440 | 1519 | 0.29 |
| 23 | Zhang et al. | 2012 | 2012 | Yunnan | Cross-sectional | Southern | … | 25 | 3.71 | 1340 | 5110 | 0.26 |
| 24 | Wang et al. | 2011 | 2010 | Yunnan | Cross-sectional | Southern | 32.2 | 18 | 0.81 | 819 | 4801 | 0.17 |
| 25 | Lian et al. | 2010 | 2010 | Hunan | Cross-sectional | Southern | … | 15 | 0.43 | 4718 | 26811 | 0.18 |
| 26 | Li et al. | 2003 | 2003 | Hunan | Cross-sectional | Southern | 43.9 | 50 | 1.51 | 208 | 1710 | 0.12 |
| 27 | Tang et al. | 2009 | 2009 | Hunan | Cross-sectional | Southern | 32.5 | 40 | 1.21 | 2128 | 5412 | 0.39 |
| 28 | Hu et al. | 2009 | 2005 | Zhejiang | Cross-sectional | Southern | … | 35 | 0.58 | 598 | 1563 | 0.38 |
| 29 | Ye et al. | 2005 | 2004 | Guangdong | Cross-sectional | Southern | 10.5 | 18 | 0.94 | 71 | 371 | 0.19 |
| 30 | Dong et al. | 2013 | 2010 | Gansu | Cross-sectional | Northern | 26.2 | 18 | 0.82 | 1100 | 3000 | 0.37 |
| 31 | Jiang et al. | 2007 | 2006 | Jiangsu | Cross-sectional | Southern | 27.8 | 15 | 1.13 | 3339 | 17308 | 0.19 |
| 32 | Qi et al. | 2013 | 2011 | Shanxi | Cross-sectional | Northern | … | 40 | 0.97 | 453 | 3020 | 0.15 |
| 33 | Zhang et al. | 2009 | 2006 | Hubei | Cross-sectional | Northern | … | 18 | 0.93 | 497 | 2257 | 0.22 |
| 34 | Yang et al. | 2010 | 2005 | Jiangsu | Cross-sectional | Southern | … | 25 | 3.88 | 1843 | 8106 | 0.23 |
| 35 | Wei et al. | 2011 | 2011 | Beijing | Cross-sectional | Northern | 43.9 | 25 | 0.00 | 530 | 2306 | 0.23 |
| 36 | Zhang et al. | 2011 | 2010 | Gansu | Cross-sectional | Northern | … | 35 | 1.31 | 5633 | 23600 | 0.24 |
| 37 | Pang et al. | 2009 | 2008 | Tianjin | Cross-sectional | Northern | 25.8 | 60 | 1.22 | 1540 | 2350 | 0.66 |
| 38 | Zhang et al. | 2008 | 2007 | Jiangsu | Cross-sectional | Southern | 25.0 | 30 | 0.73 | 429 | 1116 | 0.38 |
| 39 | Li et al. | 2004 | 2002 | Shandong | Cross-sectional | Northern | 8.9 | 35 | 1.09 | 21248 | 81509 | 0.26 |
| 40 | Zhang et al. | 2006 | 2005 | Hubei | Cross-sectional | Northern | 32.0 | 35 | 0.72 | 494 | 1523 | 0.32 |
| 41 | Zhang et al. | 2009 | 2008 | Zhejiang | Cross-sectional | Southern | 41.6 | 35 | 1.01 | 238 | 1576 | 0.15 |
| 42 | Zhang et al. | 2010 | 2006 | Shenyang | Cross-sectional | Northern | … | 15 | 1.03 | 375 | 1799 | 0.21 |
| 43 | Li et al. | 2011 | 2010 | Shenyang | Cross-sectional | Northern | 49.8 | 18 | 0.92 | 205 | 724 | 0.28 |
| 44 | Zheng et al. | 2011 | 2008 | Shenyang | Cross-sectional | Northern | 32.4 | 18 | 0.77 | 942 | 3869 | 0.24 |
| 45 | Guo et al. | 2007 | 2004 | Shanghai | Cross-sectional | Southern | … | 40 | 0.73 | 1772 | 5244 | 0.34 |
| 46 | Tang et al. | 2011 | 2010 | Shanghai | Cross-sectional | Southern | … | 60 | 0.73 | 1946 | 3620 | 0.54 |
| 47 | Yin et al. | 2009 | 2008 | Shaodong | Cross-sectional | Northern | … | 18 | 1.02 | 1083 | 4352 | 0.25 |
| 48 | Zhong et al. | 2010 | 2008 | Shandong | Cross-sectional | Northern | … | 15 | 0.70 | 2470 | 8797 | 0.28 |
| 49 | Qu et al | 2011 | 2009 | Hubei | Cross-sectional | Northern | 35.6 | 35 | 0.70 | 3522 | 9618 | 0.37 |
| 50 | Pei et al. | 2013 | 2010 | Zhejiang | Cross-sectional | Southern | … | 20 | 0.96 | 1271 | 6523 | 0.19 |
| 51 | Chen et al. | 2010 | 2010 | Shandong | Cross-sectional | Northern | … | 16 | 0.72 | 3295 | 9933 | 0.33 |
| 52 | Wan et al. | 2012 | 2011 | Jiangsu | Cross-sectional | Southern | … | 60 | 0.87 | 448 | 1122 | 0.40 |
| 53 | Liu et al. | 2009 | 2008 | Shandong | Cross-sectional | Northern | … | 35 | 1.08 | 1357 | 3997 | 0.34 |
| 54 | Yang et al. | 2010 | 2009 | Guangdong | Cross-sectional | Southern | 25.9 | 18 | 0.95 | 215 | 2639 | 0.08 |
| 55 | Zhang et al. | 2013 | 2012 | Shandong | Cross-sectional | Northern | … | 20 | 0.96 | 344 | 1615 | 0.21 |
| 56 | Zhang et al. | 2011 | 2007 | Ningxia | Cross-sectional | Northern | … | 18 | 0.93 | 163 | 686 | 0.24 |
| 57 | Gong et al. | 2011 | 2008 | Ningxia | Cross-sectional | Northern | … | 18 | 0.75 | 870 | 4941 | 0.18 |
| 58 | Chen et al. | 2012 | 2011 | Zhejiang | Cross-sectional | Southern | 26.1 | 35 | 0.78 | 433 | 1704 | 0.25 |
| 59 | Zhu et al. | 2011 | 2008 | Anhui | Cross-sectional | Northern | … | 16 | 0.65 | 425 | 2786 | 0.15 |
| 60 | Wang et al. | 2010 | 2008 | Jiangsu | Cross-sectional | Southern | 20.4 | 22 | 0.60 | 1597 | 9783 | 0.16 |
| 61 | Hu et al. | 2011 | 2011 | Jiangxi | Cross-sectional | Southern | 58.4 | 35 | 0.85 | 223 | 842 | 0.26 |
| 62 | Cui et al. | 2011 | 2010 | Henan | Cross-sectional | Northern | … | 25 | 0.72 | 209 | 1109 | 0.19 |
| 63 | Duan et al. | 2005 | 2004 | Sichuan | Cross-sectional | Southern | 19.3 | 35 | 0.96 | 131 | 790 | 0.17 |
| 64 | Yang et al. | 2005 | 2004 | Shandong | Cross-sectional | Northern | 21.0 | 35 | 0.92 | 578 | 2049 | 0.28 |
| 65 | Zhao et al. | 2012 | 2011 | Yunnan | Cross-sectional | Southern | … | 35 | 0.73 | 1525 | 5298 | 0.29 |
| 66 | Diao et al. | 2010 | 2009 | Liaoning | Cross-sectional | Northern | … | 35 | 0.79 | 1772 | 5122 | 0.35 |
| 67 | Xun et al. | 2008 | 2004 | Liaoning | Cross-sectional | Northern | … | 60 | 1.19 | 6060 | 10065 | 0.60 |
| 68 | Xing et al. | 2010 | 2008 | Liaoning | Cross-sectional | Northern | … | 35 | 0.95 | 62337 | 152981 | 0.41 |
| 69 | Wang et al. | 2010 | 2008 | Liaoning | Cross-sectional | Northern | 62.9 | 35 | … | 11321 | 30200 | 0.37 |
| 70 | Chen et al. | 2010 | 2008 | Liaoning | Cross-sectional | Northern | … | 35 | 0.76 | 8563 | 22559 | 0.38 |
| 71 | Yang et al. | 2008 | 2008 | Liaoning | Cross-sectional | Northern | 30.0 | 15 | 0.69 | 1935 | 8372 | 0.23 |
| 72 | Zhao et al. | 2009 | 2007 | Jiangxi | Cross-sectional | Southern | … | 35 | 0.77 | 986 | 1927 | 0.51 |
| 73 | Chen et al. | 2011 | 2007 | Shandong | Cross-sectional | Northern | … | 20 | … | 928 | 15213 | 0.06 |
| 74 | Yao et al. | 2008 | 2007 | Shandong | Cross-sectional | Northern | 26.2 | 35 | 1.03 | 2896 | 12873 | 0.22 |
| 75 | Zhang et al. | 2013 | 2012 | Jiangsu | Cross-sectional | Northern | … | 20 | 0.67 | 1437 | 4568 | 0.31 |
| 76 | Cai et al. | 2008 | 2005 | Yunnan | Cross-sectional | Southern | … | 45 | 0.95 | 1850 | 6006 | 0.31 |
| 77 | Li et al. | 2012 | 2011 | Liaoning | Cross-sectional | Northern | 38.2 | 15 | 0.90 | 2149 | 11682 | 0.18 |
| 78 | Sun et al. | 2008 | 2005 | Liaoning | Cross-sectional | Northern | … | 35 | 0.00 | 8948 | 23178 | 0.39 |
| 79 | Li et al. | 2010 | 2009 | Shandong | Cross-sectional | Northern | … | 40 | 0.75 | 553 | 1491 | 0.37 |
| 80 | Zhou et al. | 2008 | 2007 | Jiangxi | Cross-sectional | Northern | … | 40 | 1.09 | 3294 | 25916 | 0.13 |
| 81 | Hu et al. | 2013 | 2011 | Jiangsu | Cross-sectional | Southern | 52.1 | 18 | 0.63 | 884 | 2417 | 0.37 |
| 82 | Miao et al. | 2005 | 2004 | Jiangsu | Cross-sectional | Southern | … | 35 | 0.96 | 6830 | 20364 | 0.34 |
| 83 | Gua et al. | 2011 | 2010 | Hebei | Cross-sectional | Northern | … | 35 | 0.60 | 3790 | 11260 | 0.34 |
| 84 | Fang et al. | 2007 | 2005 | Guangdong | Cross-sectional | Southern | … | 15 | 0.63 | 203 | 1208 | 0.17 |
| 85 | Song et al. | 2010 | 2010 | Zhejiang | Cross-sectional | Southern | 13.4 | 16 | 0.65 | 425 | 2786 | 0.15 |
| 86 | Shu et al. | 2013 | 2012 | Zhejiang | Cross-sectional | Southern | … | 30 | 0.65 | 526 | 1341 | 0.39 |
| 87 | Yang et al. | 2011 | 2007 | Hunan | Cross-sectional | Southern | … | 30 | 0.91 | 762 | 3252 | 0.23 |
| 88 | Yuan et al. | 2009 | 2006 | Shanxi | Cross-sectional | Northern | … | 25 | 0.95 | 301 | 1256 | 0.24 |
| 89 | Yang et al. | 2007 | 2006 | Neimenggu | Cross-sectional | Northern | … | 35 | 0.54 | 665 | 2029 | 0.33 |
| 90 | Zhang et al. | 2006 | 2004 | Shandong | Cross-sectional | Northern | … | 50 | 0.77 | 763 | 2575 | 0.30 |
| 91 | Chen et al. | 2008 | 2007 | Henan | Cross-sectional | Northern | … | 18 | 0.71 | 252 | 984 | 0.26 |
| 92 | Han et al. | 2009 | 2007 | Henan | Cross-sectional | Northern | 20.2 | 18 | 0.65 | 6213 | 20194 | 0.31 |
| 93 | Chen et al. | 2013 | 2012 | Hebei | Cross-sectional | Northern | 55.8 | 35 | 0.66 | 1516 | 2532 | 0.60 |
| 94 | Sun et al. | 2008 | 2007 | Hebei | Cross-sectional | Northern | … | 15 | 0.67 | 2379 | 8526 | 0.28 |
| 95 | Yao et al. | 2005 | 2004 | Anhui | Cross-sectional | Northern | … | 15 | 1.09 | 754 | 5028 | 0.15 |
| 96 | Song et al. | 2008 | 2006 | Guizhou | Cross-sectional | Southern | 79.1 | 25 | 0.74 | 312 | 1468 | 0.21 |
| 97 | Xin et al. | 2011 | 2008 | Guangxi | Cross-sectional | Southern | … | 18 | 1.02 | 3225 | 21985 | 0.15 |
| 98 | Zhao et al. | 2012 | 2010 | Guangxi | Cross-sectional | Southern | … | 35 | 0.90 | 2486 | 5591 | 0.44 |
| 99 | Li et al. | 2007 | 2006 | Guangxi | Cross-sectional | Southern | … | 15 | 1.03 | 633 | 4031 | 0.16 |
| 100 | Zhao et al | 2013 | 2011 | Gansu | Cross-sectional | Northern | 34.0 | 18 | 1.00 | 648 | 2224 | 0.29 |
| 101 | Wu et al. | 2009 | 2005 | Gansu | Cross-sectional | Northern | … | 35 | 1.31 | 5673 | 23600 | 0.24 |
| 102 | Jiang et al. | 2006 | 2005 | Hubei | Cross-sectional | Northern | … | 20 | 1.10 | 423 | 1641 | 0.26 |
| 103 | Lu et al. | 2008 | 2004 | Zhejiang | Cross-sectional | Southern | … | 35 | 0.74 | 411 | 1931 | 0.21 |
| 104 | Deng et al. | 2010 | 2009 | Guangdong | Cross-sectional | Southern | 45.0 | 35 | 0.77 | 946 | 2132 | 0.44 |
| 105 | Ye et al. | 2004 | 2004 | Anhui | Cross-sectional | Northern | 42.7 | 18 | 0.55 | 123 | 709 | 0.17 |
| 106 | Mao et al. | 2005 | 2004 | Jiangsu | Cross-sectional | Southern | 28.1 | 25 | 0.77 | 2740 | 8683 | 0.32 |
| 107 | Hu et al. | 2008 | 2006 | Zhejiang | Cross-sectional | Southern | … | 18 | 0.76 | 599 | 4977 | 0.12 |
| 108 | Chen et al. | 2011 | 2010 | Jiangsu | Cross-sectional | Southern | 9.7 | 35 | 0.94 | 597 | 2831 | 0.21 |
| 109 | Li et al. | 2006 | 2005 | Beijing | Cross-sectional | Northern | 56.2 | 35 | 0.75 | 729 | 1364 | 0.53 |
| 110 | Wang et al. | 2011 | 2010 | Beijing | Cross-sectional | Northern | 86.7 | 15 | 0.85 | 1098 | 5310 | 0.21 |
| 111 | Xie et al. | 2013 | 2011 | Beijing | Cross-sectional | Northern | 52.6 | 25 | 0.30 | 391 | 2076 | 0.19 |
| 112 | Li et al. | 2008 | 2005 | Beijing | Cross-sectional | Northern | … | 30 | 0.79 | 1216 | 3653 | 0.33 |
| 113 | Zhang et al. | 2010 | 2009 | Beijing | Cross-sectional | Northern | 61.6 | 18 | 0.55 | 2126 | 5227 | 0.41 |
| 114 | Xing et al. | 2007 | 2007 | Neimenggu | Cross-sectional | Northern | … | 15 | 0.20 | 86 | 830 | 0.10 |
| 115 | Guo et al. | 2011 | 2006 | Hebei | Cross-sectional | Northern | … | 35 | 0.91 | 1888 | 4227 | 0.45 |
| 116 | Wang et al. | 2007 | 2006 | Anhui | Cross-sectional | Northern | … | 60 | … | 603 | 1422 | 0.42 |
| 117 | Huang et al. | 2012 | 2011 | Zhejiang | Cross-sectional | Southern | … | 16 | 0.90 | 522 | 6500 | 0.08 |
| 118 | Wei et al. | 2009 | 2008 | Anhui | Cross-sectional | Southern | … | 60 | 1.22 | 406 | 751 | 0.54 |
| 119 | He et al. | 2013 | 2011 | Anhui | Cross-sectional | Northern | … | 30 | 0.81 | 157 | 960 | 0.16 |
| 120 | Wang et al. | 2013 | 2010 | Anhui | Cross-sectional | Southern | … | 18 | 1.03 | 307 | 2546 | 0.12 |
| 121 | Wan et al. | 2007 | 2006 | Anhui | Cross-sectional | Northern | … | 35 | 0.92 | 2525 | 9978 | 0.25 |
| 122 | Zhang et al. | 2007 | 2005 | Anhui | Cross-sectional | Northern | … | 30 | 0.85 | 2304 | 9280 | 0.25 |
| 123 | Huang et al. | 2011 | 2010 | Fujian | Cross-sectional | Southern | 22.2 | 18 | 1.36 | 1931 | 5350 | 0.36 |
| 124 | Dong et al | 2007 | 2007 | Liaoning | Cross-sectional | Northern | 20.3 | 35 | 1.02 | 10862 | 29970 | 0.36 |
