# Supplementary material for: Prevalence of Hypertension in Rural Areas of China: A Meta-Analysis of Published Studies
Source: PLoS One. 2014 Dec 18;9(12):e115462. doi: 10.1371/journal.pone.0115462 (PMC4270770; doi:10.1371/journal.pone.0115462)
Supplement: S2 Table — Characteristic of Studies on the Prevalence of hypertension (continued Table S1). (DOC) [file pone.0115462.s002.doc]

**Table S2 Characteristic of Studies on the Prevalence of hypertension (Continued Table S1)**

| **NO.** | **First author** | **Response rate (%)** | **Sample selection method** | **Sample source** | **Diagnostic criteria** | **Method of measurement** | **Quality**  **score** |
| --- | --- | --- | --- | --- | --- | --- | --- |
| 1 | Yuan et al. | 98.4 | Clustered randomized sampling | Stratified general population | 1996,WHO | Mean value from 3 times | 8 |
| 2 | Lin et al. | 100.0 | Clustered randomized sampling | Stratified general population | 2004,China | N/A | 7 |
| 3 | Zhang et al. | 100.0 | Clustered randomized sampling | Community population | China | Mean value from 3 times | 9 |
| 4 | Jie et al. | 100.0 | Whole sample | One district population | China | Mean value from 3 times | 9 |
| 5 | Zhang et al. | 72.99 | Whole sample | One district population | N/A | N/A | 7 |
| 6 | Lin et al. | 96.5 | Clustered randomized sampling | Community population | WHO | Mean value from 3 times | 9 |
| 7 | Zhang et al. | 100.0 | Clustered randomized sampling | Stratified general population | WHO | Mean value from 3 times | 9 |
| 8 | Yu et al. | 100.0 | Whole sample | Community population | 2004,China | Mean value from 3 times | 9 |
| 9 | Sun et al. | 100.0 | Clustered randomized sampling | Stratified general population | WHO | N/A | 7 |
| 10 | Liu et al. | 99.5 | Clustered randomized sampling | General population | 1999,WHO | N/A | 7 |
| 11 | Zhou et al. | 100.0 | Clustered randomized sampling | Stratified general population | China | N/A | 8 |
| 12 | Yu et al. | 100.0 | Clustered randomized sampling | Stratified general population | China | Mean value from 2 times | 9 |
| 13 | He et al. | 99.7 | Clustered randomized sampling | One district population | N/A | Mean value from 3 times | 8 |
| 14 | Zhong et al. | 100.0 | Clustered randomized sampling | Community population | 2005,China | Mean value from 3 times | 9 |
| 15 | Yu et al. | 100.0 | Clustered randomized sampling | General population | 2005,China | Mean value from 3 times | 9 |
| 16 | Li et al. | 94.6 | Clustered randomized sampling | Stratified general population | 2005,China | Mean value from 2 times | 9 |
| 17 | Zhou et al. | 100.0 | Clustered randomized sampling | Stratified general population | 2005,China | Mean value from 3 times | 8 |
| 18 | Xia et al. | 95.5 | Clustered randomized sampling | Stratified general population | 1999,WHO | Mean value from 3 times | 6 |
| 19 | Zheng et al. | 100.0 | Whole sample | Population in health system | N/A | N/A | 7 |
| 20 | Su et al. | 100.0 | Whole sample | One district population | 2005,China | Mean value from 2 times | 10 |
| 21 | Fan et al. | 100.0 | Whole sample | One district population | China | N/A | 7 |
| 22 | Tao et al. | 98.5 | Clustered randomized sampling | Stratified general population | WHO | Mean value from 3 times | 8 |
| 23 | Zhang et al. | 90.79 | Clustered randomized sampling | Stratified general population | China | Mean value from 2 times | 9 |
| 24 | Wang et al. | 98.2 | Clustered randomized sampling | Stratified general population | China | N/A | 8 |
| 25 | Lian et al. | N/A | Whole sample | One district population | 2005,China | Mean value from 2 times | 7 |
| 26 | Li et al. | 85.5 | Clustered randomized sampling | Stratified general population | China | Mean value from 2 times | 8 |
| 27 | Tang et al. | 92.2 | Clustered randomized sampling | One district population | 2005,China | Mean value from 3 times | 9 |
| 28 | Hu et al. | 100.0 | Clustered randomized sampling | General population | WHO | Mean value from 2 times | 8 |
| 29 | Ye et al. | 100.0 | Clustered randomized sampling | Stratified general population | WHO | Mean value from 3 times | 6 |
| 30 | Dong et al. | 100.0 | Clustered randomized sampling | Stratified general population | WHO | Mean value from 2 times | 8 |
| 31 | Jiang et al. | 86.5 | Clustered randomized sampling | One district population | 1999,WHO | Mean value from 2 times | 8 |
| 32 | Qi et al. | 100.0 | Whole sample | One district population | WHO | Mean value from 2 times | 8 |
| 33 | Zhang et al. | 100.0 | Clustered randomized sampling | General population | WHO | Mean value from 3 times | 8 |
| 34 | Yang et al. | 99.7 | Clustered randomized sampling | General population | 2004,China | Mean value from 3 times | 9 |
| 35 | Wei et al. | 100.0 | Whole sample | One district population | 2007,WHO | N/A | 7 |
| 36 | Zhang et al. | 100.0 | Whole sample | One district population | 2005,China | Mean value from 3 times | 9 |
| 37 | Pang et al. | 100.0 | Clustered randomized sampling | General population | China | Mean value from 3 times | 9 |
| 38 | Zhang et al. | 100.0 | Clustered randomized sampling | General population | WHO | Mean value from 3 times | 9 |
| 39 | Li et al. | 100. | Clustered randomized sampling | General population | 1999,China | N/A | 7 |
| 40 | Zhang et al. | 91.1 | Whole sample | Population in health system | China | Mean value from 2 times | 9 |
| 41 | Zhang et al. | 100.0 | Clustered randomized sampling | General population | 1999,China | Mean value from 3 times | 9 |
| 42 | Zhang et al. | 100.0 | Clustered randomized sampling | General population | China | Mean value from 3 times | 8 |
| 43 | Li et al. | 92.6 | Whole sample | One district population | 1999,WHO | Mean value from 3 times | 7 |
| 44 | Zheng et al. | N/A | Clustered randomized sampling | General population | WHO | Mean value from 3 times | 6 |
| 45 | Guo et al. | 85.9 | Clustered randomized sampling | General population | China | Mean value from 3 times | 7 |
| 46 | Tang et al. | 100.0 | Whole sample | Population in health system | 2005,China | Mean value from 3 times | 8 |
| 47 | Yin et al. | 90.6 | Clustered randomized sampling | Stratified general population | 1999,WHO | Mean value from 3 times | 9 |
| 48 | Zhong et al. | 91.4 | Clustered randomized sampling | Stratified general population | 2005,China | Mean value from 3 times | 8 |
| 49 | Qu et al | 81.3 | Clustered randomized sampling | General population | China | Mean value from 3 times | 8 |
| 50 | Pei et al. | 100.0 | Clustered randomized sampling | General population | 2010,China | Mean value from 3 times | 9 |
| 51 | Chen et al. | 96.9 | Whole sample | One district population | 2005,China | Mean value from 3 times | 7 |
| 52 | Wan et al. | 100.0 | Whole sample | Population in health system | 2009,China | Mean value from 2 times | 6 |
| 53 | Liu et al. | 100.0 | Clustered randomized sampling | General population | 2005,China | Mean value from 3 times | 7 |
| 54 | Yang et al. | 100.0 | Whole sample | One district population | China | Mean value from 3 times | 8 |
| 55 | Zhang et al. | 80.7 | Clustered randomized sampling | Stratified general population | China | Mean value from 3 times | 8 |
| 56 | Zhang et al. | 100.0 | Clustered randomized sampling | General population | China | N/A | 7 |
| 57 | Gong et al. | 100.0 | Clustered randomized sampling | General population | 2005,China | Mean value from 3 times | 6 |
| 58 | Chen et al. | 100.0 | Clustered randomized sampling | Stratified general population | 1999,WHO | Mean value from 3 times | 7 |
| 59 | Zhu et al. | 100.0 | Clustered randomized sampling | General population | China | N/A | 7 |
| 60 | Wang et al. | 100.0 | Whole sample | Population in health system | China | Mean value from 3 times | 6 |
| 61 | Hu et al. | 100.0 | Clustered randomized sampling | General population | China | N/A | 7 |
| 62 | Cui et al. | 100.0 | Clustered randomized sampling | General population | 2005,China | N/A | 7 |
| 63 | Duan et al. | 80.0 | Stratified randomized sampling | General population | 1999,WHO | Mean value from 3 times | 8 |
| 64 | Yang et al. | 100.0 | Clustered randomized sampling | General population | 1999,WHO | Mean value from 3 times | 8 |
| 65 | Zhao et al. | 100.0 | Whole sample | Population in health system | 2005,China | Mean value from 3 times | 6 |
| 66 | Diao et al. | 98.9 | Clustered randomized sampling | General population | China | N/A | 8 |
| 67 | Xun et al. | 100.0 | Clustered randomized sampling | Stratified general population | 2005,China | Mean value from 3 times | 9 |
| 68 | Xing et al. | 96.8 | Whole sample | One district population | 2005,China | Mean value from 3 times | 9 |
| 69 | Wang et al. | 100.0 | Clustered randomized sampling | General population | China | Mean value from 3 times | 9 |
| 70 | Chen et al. | 100.0 | Clustered randomized sampling | Stratified general population | WHO | Mean value from 3 times | 9 |
| 71 | Yang et al. | 81.1 | Clustered randomized sampling | General population | WHO | Mean value from 3 times | 7 |
| 72 | Zhao et al. | 95.6 | Whole sample | One district population | WHO | Mean value from 3 times | 7 |
| 73 | Chen et al. | 90.4 | Clustered randomized sampling | General population | WHO | Mean value from 3 times | 8 |
| 74 | Yao et al. | 100.0 | Clustered randomized sampling | General population | WHO | Mean value from 3 times | 8 |
| 75 | Zhang et al. | 100.0 | Whole sample | One district population | 2010,China | Mean value from 3 times | 9 |
| 76 | Cai et al. | 100.0 | Clustered randomized sampling | General population | WHO | Mean value from 2 times | 8 |
| 77 | Li et al. | 100.0 | Whole sample | One district population | 2009,WHO | Mean value from 2 times | 9 |
| 78 | Sun et al. | 100.0 | Whole sample | One district population | WHO | Mean value from 2 times | 8 |
| 79 | Li et al. | 99.1 | Clustered randomized sampling | General population | 2005,China | Mean value from 3 times | 8 |
| 80 | Zhou et al. | 100.0 | Whole sample | One district population | China | Mean value from 3 time | 7 |
| 81 | Hu et al. | 100.0 | Clustered randomized sampling | General population | China | Mean value from 1 time | 7 |
| 82 | Miao et al. | 100.0 | Clustered randomized sampling | General population | China | Mean value from 3 time | 8 |
| 83 | Gua et al. | 100.0 | Clustered randomized sampling | General population | 2005,China | Mean value from 3 times | 9 |
| 84 | Fang et al. | 100.0 | Whole sample | One district population | 2005,China | Mean value from 3 times | 8 |
| 85 | Song et al. | 98.6 | Clustered randomized sampling | General population | 1999,WHO | Mean value from 3 times | 7 |
| 86 | Shu et al. | 89.4 | Clustered randomized sampling | General population | China | Mean value from 2 times | 8 |
| 87 | Yang et al. | 96.5 | Clustered randomized sampling | General population | 1999,WHO | Mean value from 3 times | 7 |
| 88 | Yuan et al. | 98.0 | Clustered randomized sampling | Stratified general population | 1999,WHO | Mean value from 3 times | 8 |
| 89 | Yang et al. | 93.0 | Whole sample | One district population | WHO | Mean value from 3 times | 9 |
| 90 | Zhang et al. | 92.2 | Clustered randomized sampling | General population | WHO | Mean value from 2 times | 7 |
| 91 | Chen et al. | 100.0 | Clustered randomized sampling | Community population | 2005,China | Mean value from 2 times | 10 |
| 92 | Han et al. | 100.0 | Clustered randomized sampling | General population | 2005,China | Mean value from 3 times | 8 |
| 93 | Chen et al. | 100.0 | Whole sample | Population in health system | 2005,China | Mean value from 3 times | 9 |
| 94 | Sun et al. | 100.0 | Clustered randomized sampling | General population | China | Mean value from 3 times | 9 |
| 95 | Yao et al. | 100.0 | Clustered randomized sampling | Stratified general population | 1999,WHO | N/A | 8 |
| 96 | Song et al. | 100.0 | Whole sample | One district population | China | Mean value from 3 times | 8 |
| 97 | Xin et al. | 100.0 | Clustered randomized sampling | General population | 2005,China | Mean value from 2 times | 9 |
| 98 | Zhao et al. | 91.2 | Clustered randomized sampling | General population | China | Mean value from 3 times | 8 |
| 99 | Li et al. | 100.0 | Clustered randomized sampling | Stratified general population | 1999,WHO | Mean value from 3 times | 8 |
| 100 | Zhao et al | 100.0 | Clustered randomized sampling | Stratified general population | WHO | Mean value from 3 times | 7 |
| 101 | Wu et al. | 100.0 | Clustered randomized sampling | Stratified general population | WHO | Mean value from 1 times | 10 |
| 102 | Jiang et al. | 91.2 | Clustered randomized sampling | General population | 1999,WHO | Mean value from 3 times | 8 |
| 103 | Lu et al. | 100.0 | Clustered randomized sampling | General population | 1999,WHO | Mean value from 3 times | 7 |
| 104 | Deng et al. | 100.0 | Clustered randomized sampling | General population | 1999,WHO | Mean value from 3 times | 8 |
| 105 | Ye et al. | 100.0 | Clustered randomized sampling | General population | 1999,WHO | Mean value from 2 times | 8 |
| 106 | Mao et al. | 85.6 | Clustered randomized sampling | General population | WHO | Mean value from 3 times | 7 |
| 107 | Hu et al. | 100.0 | Whole sample | One district population | 2005,China | Mean value from 3 times | 9 |
| 108 | Chen et al. | 100.0 | Clustered randomized sampling | Community population | China | Mean value from 3 times | 6 |
| 109 | Li et al. | 100.0 | Clustered randomized sampling | General population | China | Mean value from 3 times | 10 |
| 110 | Wang et al. | 96.8 | Clustered randomized sampling | General population | China | Mean value from 3 times | 8 |
| 111 | Xie et al. | 99.4 | Clustered randomized sampling | General population | China | Mean value from 3 times | 8 |
| 112 | Li et al. | 100.0 | Clustered randomized sampling | Community population | 2005,China | Mean value from 3 times | 9 |
| 113 | Zhang et al. | 100.0 | Whole sample | One district population | 2005,China | Mean value from 3 times | 7 |
| 114 | Xing et al. | 100.0 | Clustered randomized sampling | General population | China | Mean value from 2 times | 6 |
| 115 | Guo et al. | 100.0 | Whole sample | One district population | China | Mean value from 3 times | 7 |
| 116 | Wang et al. | N/A | Clustered randomized sampling | General population | WHO | Mean value from 3 times | 9 |
| 117 | Huang et al. | 100.0 | Whole sample | One district population | WHO | Mean value from 3 times | 8 |
| 118 | Wei et al. | 100.0 | Whole sample | One district population | 2005,China | Mean value from 3 times | 8 |
| 119 | He et al. | 91.9 | Clustered randomized sampling | General population | 2005,China | Mean value from 3 times | 8 |
| 120 | Wang et al. | 100.0 | Clustered randomized sampling | General population | 1999,WHO | Mean value from 2 times | 7 |
| 121 | Wan et al. | 100.0 | Clustered randomized sampling | General population | 2005,China | Mean value from 3 times | 8 |
| 122 | Zhang et al. | 100.0 | Clustered randomized sampling | Community population | 1999,WHO | Mean value from 2 times | 8 |
| 123 | Huang et al. | 93.9 | Clustered randomized sampling | Stratified general population | WHO | Mean value from 3 times | 9 |
| 124 | Dong et al | 99.2 | Whole sample | One district population | WHO | Mean value from 3 times | 9 |
