# Supplementary material for: Prevalence of Hypertension in Rural Areas of China: A Meta-Analysis of Published Studies
Source: PLoS One. 2014 Dec 18;9(12):e115462. doi: 10.1371/journal.pone.0115462 (PMC4270770; doi:10.1371/journal.pone.0115462)
Supplement: S1 Diagram — PRISMA Flow Diagram. (DOC) [file pone.0115462.s004.doc]

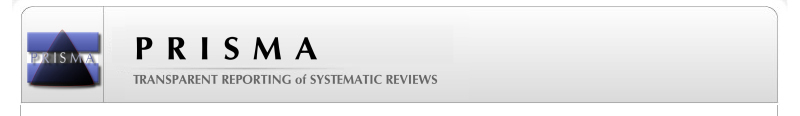
**PRISMA 2009 Flow Diagram**

**Screening**

**Included**

**Eligibility**

**Identification**

Records identified through database searching
(n = 1619)

Additional records identified through other sources
(n =0)

Records after duplicates removed
(n = 1015)

Records screened
(n = 1015)

Records excluded
(n =762)

Full-text articles assessed for eligibility
(n = 253)

Full-text articles excluded, with reasons
(n = 129)

Studies included in qualitative synthesis
(n =124 )

Studies included in quantitative synthesis (meta-analysis)
(n =124 )
