# Supplementary material for: Prevalence of Hypertension in Rural Areas of China: A Meta-Analysis of Published Studies
Source: PLoS One. 2014 Dec 18;9(12):e115462. doi: 10.1371/journal.pone.0115462 (PMC4270770; doi:10.1371/journal.pone.0115462)
Supplement: S1 References — References included in the meta-analysis. (DOC) [file pone.0115462.s005.doc]

**Supplementary 1 references included in the meta-analysis**

1. Yuan JH, Yang JZ, Guo CZ, Chai FM. Analysis on relation between dietary intakes and hypertension among 1256 rural residents in Shanxi Province. Pract Prev Med. 2009; 16(1): 79-81.

2. Lin YL, Yu M, Hong B. Analysis of health check-up result from 1853 farmers. Modern Prev Med. 2008; 35(6): 1128-1129.

3. Zhang JT, Wang YL, Zhang YX, Chen C, Shen HB. Analysis of the prevalence of hypertension and its risk factors among rural older population in Wujin district of Changzhou City from 2004 to 2005. Pre. Med. Trib. 2008; 14(6): 489-491.

4. Xie HX, Song GD, Zhang H, Tian HG. Analysis on blood pressure of rural residents in Tianjin in 2004. Chin J Prev. Contr. No-commum. Dis. 2007; 15(2): 97-100.

5. Zhang W, Fang M. The investigation of major non-communicable disease of farmer in Fuchen district of Mianyang City in 2006. Pre Med Trib. 2008; 14(10): 902-903.

6. Li Y, Lin SX, Li XD, Yin P. Investigation on the prevalence of hypertension in rural residents aged over 20 in Liaoshan district of Qingdao in 2006. Pre Med Trib. 2009; 15(2): 143-144.

7. Zhang AH, Zhang SW. Investigation on the prevalence of hypertension in rural residents aged over 18 in Zoucheng Ctiy in 2006. Prev Med Trib. 2009; 15(8): 720-721.

8. Yu YX, Yang XC. Investigation of check-up among rural residents in 2008.Med J Chin People Health. 2009; 21(19): 2364-2366.

9. Sun Z, Zheng L, Detrano R, et al. The accelerating epidemic of hypertension among rural Chinese women: results from Liaoning Province. Am J Hypertens, 2008, 21(7):784-788.

10. Liu WF. Assocation between obesity and hypertension among rural residents of Nanyue County in 2010. Prac Prev Med. 2011; 18(7):1362-1363.

11. Zhou YX. Survey on hypertension among rural residents in Huangcheng Township, Weishan County. Pre Med Trib. 2011; 17(10): 909-913.

12. Yu QH. Investigation on the prevalence of chronic non-communicable disease in rural residents aged over 18 in Yutai County. World Health Dig Med. Periodieal. 2011; 8(35): 13-14.

13. He F, Qiu M, Chen WG, Xu JJ, Zhu HB, et al. Major chronic diseases and risk factors survey of rural residents in Yandu District, 2011. Chin J dis Control Prev. 2012; 16(7): 560-563.

14. Zhong DC, Cao WZ, Zhang T, Yan H, Xiong Z. Analysis of the prevalence of hypertension and its risk factors among rural population in Zigong City. Sichuan Med J. 2009; 30(7): 1056-1058.

15. Yu SQ, Zhang QQ, Zhang SL, Zhou WZ, Gao GY. Analysis of the prevalence of hypertension and its risk factors among rural population in Zibo City. Chin Prev Med. 2010; 11(11): 1158-1160.

16. Li XS, Wang P. Relationship between unhealthy lives habits among rural population in Zhuzhou city. Chin J Prac Nur. 2013; 5(29): 185-186.

17. Zhou BQ, Li DY, Liang XD. Logistic regression analysis on influencing factors of hypertension in rural areas of Zhuhai. Prac Prev Med. 2011; 18(7): 1200-1202.

18. Xia YY, Li G, Ding XB, Mao DQ, QI L, et al. Prevalence of hypertension and its associated factors among rural residents in Chongqing. J Trop Med. 2013 13(3): 350-353.

19. Zheng ZH, Liang PS, Kuang ZP. Study on prevalence and related factors of chronic diseases among adults in rural area of Zhongshan city. China Chlin Prac Med. 2010; 4(1): 219-220.

20. Su MM, Tian N, Li ST, Ying XH, Fu CW, et al. Community-based cross-sectional survey on prevalence and awareness proportion of hypertension among rural adults in Yuhuan County of Zhejiang province, China. Fudan Univ J Med. 2013; 40(5): 556-561.

21. Fan JD. Investigation of prevalence of hypertension among rural residents in Zhejiang province. Chin Prac J Rural Doct. 2013; 20(2):128-132.

22. Tao J, Cai L, Yang M, Zhao KY, Tang PF. Analysisy of prevalence and economic burden of hypertension in a rural area of Yunnan province. Modern Prev Med. 2009; 36(23): 4456-4457.

23. Zhang M, Liu YC, Meng Y, Yang YL, Dong CQ. Hypertension prevalence on rural mountain areas of Yunnan province. Chin Gene Prac. 2012; 15(9): 3041-3044.

24. Wang KW, Cai L, Shu ZK, Dong J, Ye YH, et al. Relationship between prevalence of cardiovascular diseases and clustering of risk factors among rural residents in Luoping county of Yunnan province. Chin J Public Health. 2011; 27(10): 1291-1292.

25. Lian SS. Prevalence of hypertension in rural residents in Ruanlin County. Jilin Med. 2010; 31(22): 3741-3742.

26. Li ZX, Gui QJ, Huang DJ. Study on prevalence and related factors of chronic diseases among adults in rural area of Yongzhou city. J Chin Physican. 2004; 5(8): 1145-1146.

27. Tang YJ, Huang SX, Sun XC. Epidemiological survey on hypertension of middle-aged and elderly people in villages of Lingling district, Yongzhou city. Prac Prev Med. 2009; 16(2): 598-600.

28. Hu YW, Hu DY, Hu B, Chen SZ. Epidemiological investigation on hypertension in Yongjia County. Chin Rural Health Service Administration. 2009; 29(12): 922-924.

29. Ye HF. Liu XM. Su YA. Zhao XB. Luo BH. Analysis of prevalence features of hypertension and influential factors in rural inhabitants in Yingfe City. Chin Trop Med. 2005; 5(5):1114-1116.

30. Dong C X, Ge P F, Ren X L, Fan HQ, Yan X. Prevalence, awareness, treatment and control of hypertension among adults in rural north-western China: a cross-sectional population survey. JOURNAL OF INTERNATIONAL MEDICAL RESEARCH. 2013, 41(4):1291-1300.

31. Sun F, Yu H, Qian XQ, Cai X, Chen YJ. Study on prevalence rate of hypertension and relevant factors among people in Yangzhou rural community. Applied J Gene Prac. 2007; 5(11): 1006-1007.

32. Qi HY, Lu JC, Liu YH, Xu XC, Li XP, et al. Analysis of epidemiological characteristics and risk factors oh hypertension in rural population in Yanan city. Chin Med Hearald. 2013; 10(1): 135-146.

33. Zhang G, Zhu CY, Zhang ZF, Duan JJ, Xia J, et al. Investigation on hypertension in rural adults in Wuhan. 2009; 20(4): 24-26.

34. Yang M. Investigation of hypertension prevalence and its factors among county folks of Wujiang Municipal. Modern Hospital. 2010; 10(9): 147-148.

35. Wei YX. Survey of hypertension, hyperlipidemia and diabetes among rural women in Tongzhou district. Prac J Cardio-cerebral pulmonary vascular. 2011; 19(10): 1677-1678.

36. Zhang TM, Gao XD, Liu YQ. The survey analysis on prevalence of hypertension and protection factor among in Tianshui rural areas residents. Chin Primary Health Care. 2011; 25(3): 42-43.

37. Peng XF, Jia LX, Zhang QZ, Guan RC, Hu D. Investigation of hypertension among elderly population in Tianjin. Chin Coal and Med J. 2009; 12(9): 1429.

38. Zhang P. Investigation of hypertension prevalence among rural residents in Taizhou. J Taizhou Polytech Institute. 2008; 8(1): 58-60.

39. Li SJ, Lin X, Zhang T, Li DH, Wang ZH, et al. A present study on hypertension population in rural areas. J Taishan Med College. 2004; 25(3): 197-199.

40. Zhang ZB, Shu CB, Luo HM, Shen CA. Investigation of hypertension prevalence and its factors among county folks of Suizhou. Chin J Prev Chronic Dis. 2006; 14(6): 429-430.

41. Zhang ZY. Epidemiology investigation of hypertension among rural aged over 35 in Songyan County. Chin Prim Health Care. 2009; 23(5): 76-77.

42. Zhang DM, Yang RP. Study on prevalence of hypertension in rural residents of Shenyang city.

Chin J Public Health. 2010; 26(4): 506-507.

43. Li Y. The Population distribution of hypertension and risk factors in countryside of Shenyang. 2011; 13(4): 228-230.

44. Zheng YT, Wang Y. Analysis of prevalence of hypertension and its risk factors in rural areas of Shenyang city. Chin Med J Metall Indus. 2011; 28(5): 573-574.

45. Guo JP, Huang JY, Huan YF, Yang YJ, Shen FY, et la. Survey of hypertension in a rural area of Shanghai City. Chin General Prac. 2007; 10(15): 1267-1271.

46. Tang JH, Wang ZP, Lin H, Fang SJ, Xu GR. Epidemiology survey of prevalence of hypertension in rural residents of Shanghai. Chin Prac J Rural Doctor. 2011; 18(4): 34-36.

47. Yin CG, Ma YX, Wang SL, Xu TL, Yan P. Epidemiology study of hypertension in rural areas of Shandong province. Prev treatment of Cardio-cerebral-vascular Dis. 2009; 9(2): 137-138.

48. Zhong GF, Zhang WM, Gao J, Xiang YZ, Wang XH. Hypertension prevalence and related factors among residents in rural of Shangdong province. Chin J Public Health. 2010; 26(11): 1345-1347.

49. Qu YL, Tan SF, Yuan SS, Chen F, Qu KY. Epidemiological characteristics and control status of hypertension in rural area of the Three Gorges. Chin J Cardiology. 2011; 39(9): 861-864.

50. Pei LP, Xu XH, Fang CF, Zhu GY, Gan ZJ. Epidemiological status and risk factors of hypertension in rural residents in Kecheng district of Quzhou city. Acta Med Uni SCi Technol Huazhong, 2013; 42(3): 351-353.

51. Chen HY, Chen MC, Li FH, Feng ZC. Investigation on the rates of awareness, treatment and control of hypertension among rural population in Qingdao mound regioin. Modern Prev Med. 2010; 37(21): 4068-4072.

52. Wan WC. Hypertension prevalence and related factors among residents aged over 60 in rural of Qidong city. Anhhui J Pre Med. 2012; 18(6): 463-464.

53. Liu LQ, Wang L. Investigation on prevalence of hypertension in rural population of Pingdu city.

Chin Heart J. 2009; 21(3): 361.

54. Yang HJ, Zhou ZH, Wang JJ. Analysis of influencing factors of hypertension in rural community. Chin J General Prac. 2010; 8(7): 896-898.

55. Zhang WL. Hypertension prevalence and related factors among residents in rural areas. Chin Health Vision. 2013; 21(4): 8.

56. Zhang Y, Liu WX, Shi ZY, Li JY, Gao YZ. Hypertension prevalence and related factors among residents in rural areas of Ji County. Ningxia Med J. 2011; 33(12): 1222-1223.

57. Gong J, Jia SB, Ma L. Prevalence of hypertension and its risk factors among rural residents in Ningxia. Chin Prev Med. 2011; 12(3): 227-229.

58. Chen L, Hao LM, Qiu XJ. Hypertension prevalence and related factors among residents in rural areas of Ningbo city. Shanghai J Prev Med. 2012; 24(12): 655-656.

59. Zhu Y, Xu XD, He Q, Wang Q. Analysis on health status and its influence factors of rural residents in Nanxun district. Modern Prev Med. 2011; 38(1): 100-103.

60. Wang X, Investigation of hypertension prevalence in rural areas of Nanjing city. Chin Foreign Med Res. 2010; 8(6): 158-159.

61. Hu LH. Association between body weight and hypertension in rural areas of Nanchang County. Med Information. 2011; 24(11): 98-99.

62. Epidemiology investigation on prevalence of hypertension and dilates in rural residents of Minchi County. Hennan J Prev Med. 2001; 22(4): 289-290.

63. Duan LH, Yue SJ, Zhou Y, Ou Y, Hu R, et al. Prevalence of hypertension and its risk factors among rural residents in Mianyang city. J Prev Med Inf. 2005; 21(1): 100-101.

64. Yang CL, Diao WX, Xu H, Yang BC, Li JY. Risk factors of primary hypertension in rural residents in north of Shandong province. J Bingzhou Vocational College. 2005; 2(1): 77-80.

65. Zhao HM. Analysis of hypertension screening among rural residents aged over 35 in Lincang city. Chin Hwalth Care Nutr. 2012(5): 343.

66. Diao WL, Zheng LQ, Xing LY, Mu HJ, Liu ZY, et al. Hypertension prevalence and its awareness medication and control among rural residents in Liaoning province. Chin Public Health. 2010; 26(5): 602-603.

67. Xun P, Gao H, Tao GZ, Sun ZQ, Zheng LQ, et al. Survey on prevalence awareness treatment and control of hypertension in the elderly rural people in Liaoning province. Pract Geriatr. 2008; 22(2): 125-127.

68. Xing LY, Diao WL, Liu ZY, Yu LY, et al. Prevalence, awareness, treatment and control of hypertension in rural adult residents of Liaoning province. Chin J Cardiol. 2010; 38(7): 652-655.

69. Wang Y, Li H, Zhou YJ. Hypertension prevalence and its factors in rural areas of Fu County. Chin Prac Med. 2010; 5(30): 261-262.

70. Chen HN, Zhang JH, Song YR, Luo YJ, Liu D, et al. Prevalence of hypertension in rural adult residents in Dawa county. Chin J Cardio Med. 2010; 15(6): 447-448.

71. Yang HY, Wang Y, Cui YQ, Fu Y, Lan YJ, et al. Epidemiology investigation of hypertension in rural areas of Benxi county. World Health Digest Med Peroi. 2008; 8(34): 46-48.

72. Zhao CQ, Zhao CS. An epidemiological survey on hypertension in rural population of a town of Lianyungang city. Chin Modern Doctor. 2009; 47(33): 97-98.

73. Chen JM. Investigation on prevalence of chronic non-communicable disease and its disease burden in rural residents in Lijin County. Chin J Coal Indus Med. 2011; 14(8): 1238-1240.

74. Yao ZF. Prevalence of hypertension among rural residents aged over 35 in Laiwu city. South Chin J Prev Med. 2008; 34(5): 49-51.

75. Zhang T. Epidemiology investigation of chronic diseases in rural areas of Hunshan city. Chin J Prev Contr Dis. 2013; 21(5): 576-578.

76. Cai L, Dong J, Bi WH, Xu CZ, Lu YC. Analysis on socioeconomic determinants of hypertension for residents in rural area of Kunming. Chin J Public Health. 2008; 24(3): 259-260.

77. Li AX, Liu XL. Hypertension prevalence and related influencing factors among rural residents in Jinzhou city. Shangdong Med. 2012; 52(34): 40-42.

78. Sun Z, Zheng L, Detrano R, Zhang DY, Zhang XG, et al. The accelerating epidemic of hypertension among rural Chinese women: results from Liaoning Province. Am J Hypertens, 2008, 21(7):784-788.

79. Li J, Tian XC, Wu WF, Qiao Q, Ning F, et al. Prevalence of hypertension and its related risk factors county daellers of Jiaonan. Med J QiLu. 2010; 25(6): 501-503.

80. Zhou YP, Zhu LP, Zhou YS. Investigation of prevalence rate and risk factor of essential hypertension among rural population aged 40-59 in Jiangxi Province. Chin Prev Med. 2008; 9(12): 1036-1039.

81. Hu XJ, Chen H, Dong YP, Chu XC, Lian YS. Epidemiological survey of hypertension among rural resident in Wujiang District of Jiangsu province. Chin Health Service Management. 2013; 5(299): 391-393.

82. Miao CX, Zhuo L, Han LC, Chen J. Epidemiological survey of hypertension among rural resident in Dongshan county of Jiangsu province. J Handan Med College. 2005; 18(2): 98-100.

83. Guo GY, Wang JX, Tian L. Investigation of hypertension prevalence among rural population aged over 35 in Jize County. Trad. Chin Med Mongolia. 2011; 10(6): 60-61.

84. Fang KF, Wu PS, Bao TC, Chen W, Wen JY, et al. Epidemiology investigation of hypertension prevalence in rural areas of Liangjin county. South Chin J Prev Med. 2007; 33(4): 29-40.

85. Song ZM, Zhou Lh, Ye XM. Investigation on prevalence of chronic non-communicable disease and its disease burden in rural residents in Nanxun County. Chin Rural Health Service Administration, 2010; 30(90): 769-771.

86. Shu CZ, Sun Y, Gao YD, Shen XH, Wang Z. Survey on prevalence of hypertension and influencing factors in rural areas of Huzhou city. Modern Prev Med. 2013; 40(19): 3629-3635.

87. Yang Q. Investigation on hypertension prevalence and treatment status in rural areas of Changde city. J Clin Res. 2011; 28(5): 974-975.

88. Yuan JH, Yang JZ, Guo CJ, Chai FM. Unconditioned Logidtic analysis of hypertension and the relationship with dietary intake among rural population in Huguan County. Prev Med Trib.2009; 15(3): 217-218.

89. Yang WX, Zhao XS, Zhou YB, Wang YX, Wang J, et al. Survey on hypertension prevalence and [electrocardiogram](javascript:void(0);) among rural population in the outskirts of Huhaohaote. Chin Clin Prac Med. 2007; 1(12): 39-41.

90. Zhang WQ, Shi YP. Survey on prevalence of hypertension among elderly rural residents in Heze City. Occupation Health. 22(12): 1377-1378.

91. Chen B, Liu JY, Sun L, Wang JF, Yang JX, et al. Prevalence and risk factors of hypertension in rural communities in Henan. Moedern Prev. Med. 2008; 35(15): 2823-2826.

92. Han B, Yu DH, Wang CJ, Ping GZ, Lu J, et al. Prevalence, awareness, treatment and control of hypertension in rural areas of Henan. J Zhengzhou Univ.2009; 44(2): 337-339.

93. Chen LZ, Ding RJ, Shi QT, Hu DY. Risk factors and prevalence of cardiovascular disease of rural residents in Xianghe of Hebei province. Chin J Cardiol. 2013; 41(10): 882-885.

94. Sun GP, Wu XS, Liu WM, Qin XH. Study on the hypertension prevalence condition of countryside inhabitants with moderate level of income in Dingzhou of Hebei. Modern Prev. Med.2008; 35(22): 4393-4396.

95. Yao H, Wang LL, Li XR, Ma EJ. Survey on the prevalence and risk factors of hypertension in Hefei rural population. Chin J Dis Control Prev. 2005; 9(1): 299-301.

96. Song FY, Xiong QJ, Li CF, You DJ. Prevalence and risk factors of hypertension among rural population of Wengan County, Guizhou Province. Prac Prev Med. 2008; 15(4): 1259-1261.

97. Xin RB, Meng GY, He L, Lu J, Huang WY, et al. Sampling survey of hypertension in Zhuang nationality in Tiandong county. Guangxi Med. 2011; 33(8): 963-965.

98. Zhao YY, Jiang X, He WZ, Huang YM, Ma Q. Analysis of epidemiology baseline investigation on hypertension in rural communities in Pubei County of Guangxi. Guanxi Med J. 2012; 34(12): 1625-1628.

99. Li RF, Li FW, Lai WJ, Huang BJ, Liu R, et al. Survey on the prevalence and risk factors of hypertension in Guangxi rural population. Guangxi Med J. 2007; 29(2): 230-232.

100. Zhao JQ. Investigation on current situation of hypertension and its influencing factors in rural population in Xihe county of Gansu province. Bull Dis Control Prev. 2013; 28(1): 27-29.

101. Epidemic status and risk factord of hypertension in rural areas of TianShui, Gansu Province. Chin J Nat Med. 2009; 11(6): 423-425.

102. Jiang RG. Survey on prevalence, knowledge and behavior of hypertension in rural areas of south Hubei. Modern Prev Med. 2006; 33(5): 824-825.

103. Lu QY, Guo GQ, Zhao QP, Guo LZ. Epidemiology investigation of hypertension in rural areas of Dongyang city. Zhejiang J Chin West Med. 2008; 18(8): 525-526.

104. Deng WB, Ye YM, Han C, Sun BL, Zheng YL, et al. Survey of hypertension prevalence among middle-aged rural residents in Dongguan city. China Clin Prac Med. 2010; 4(3): 211-213.

105. Ye ZW, Xiang QY. Surveillance report on nutritional and health conditions of the rural residents in Juchao District, Chaohu city. Anhui J Prev Med. 2011; 17(3): 190-191.

106. Mao HD, Wu JF, Zhou YH, Jiang WP, Wang YL. Epidemiology survey on the status of prevalence of hypertension among rural areas in Changzhou. Chin J Dis Control Prev. 2005; 9(5):406-408.

107. Hu HH. Survey on the prevalence, Knowledge, treatment and control in rural area of Changshan County. Prev and Treatment Cardio-Cerabral Vascular Dis.2008; 8(3): 195-196.

108. Hypertension prevalence of rural population in resettlement area. China Modern Doctor. 2011; 49(11): 92-93.

109. Li YD, Gu XJ, Wu T, Zhang SJ, Li W. Cross-sectional study on the prevalence of hypertension and its related risk factors in rural Chinese aged 35-70 in Shunyi District， Beijing. Chin J Prev Contr Chron Non-commun Dis. 2006; 14(4): 245-247.

110. Wang Yu, Zhang Y. Survey on knowledge of hypertension and diabetes. Chin J Public Health. 2011; 27(6): 787-788.

111. Xie XM. Study on the prevalenc of hypertension and the therapeutic effect in rural areas of Miyun, Beijing. Capital J of Public Health. 2013; 7(5): 211-213.

112. Li SJ, Zhang XY, Zheng XB, Zheng SH. Investigation status of hypertension in rural residents aged over 30 years in Jingzhuang Town, Yangqing District. Beijing. Chin J Prev Contr Chron Non-commun Dis.2008; 16(5): 490-492.

113. Zhang LJ, He SP, Liu YF, Liu CX. Analysis on results of physical examination among rural residents Huairou district of Beijing city. Occup and Health. 2010; 26(16): 1872-1874.

114. Xing YQ. Li W, Zhang GJ. Survey on prevalence of hypertension in rural areas of Batou city. Baotou Med Univ J. 2007; 4(24): 353-354.

115. Guo Y, Hao YM, Ma JX. Epidemiology survey on prevalence of hypertension among rural residents in the east part of Bazhou city. Hebei Med J. 2011; 33(3): 443.

116. Wang XY, Tao XY, Hu CL, Yan ZY, Li YC. Epidemiological investigation on hypertension among the aged female population in a rural community of Anhui province. Chin Rural Health Service Administration. 2007; 27(12): 914-915.

117. Huang YY. Analysis of checkup results from 6500 rural residents. Seek Med and Ask the Med. 2010; 10(3): 611.

118. Wei HC. Zhang WJ. Jiu PX. Wang M, Pan JY, et al. Prevalence and influencing factors of hypertension in elderly rural residents of Anhui province. Chin J Public Health. 2009; 25(8): 962-964.

119. He ZF, Ding H, Li XF, Sun X. Investigation on the rural residents’ knowledge of hypertension and their health behaviors in Anhui Province. Med and Soci. 2013, 26(8): 51-53.

120. Wang YM. Zhu JM, Li BK, Hu GH. Investigation of epidemic characters of hypertension disease of rural residents in Anhui province. J Benbu Med Coll. 2013; 38(3): 321-323.

121. Wan YH. Hu CL, Yan ZY, Tao XY, Li YC, et al. Research on prevalence and risk factors of hypertension among residents in a certain village and township community. Modern Prev Med. 2007; 34(18): 3410-3412.

122.Dan X, Hu CL, Yan ZY, Tao XY, Li YC. Investigation on cognition and prevalence of hypertension in a rural area. Chin J Public Health. 2007; 23(7): 864-865.

123. Huang JP, Zhang W, Li XH, Zhou JX, Gao Y, et al. Analysis of the prevalence and risk factors oh hypertension in the She population in Fujian, China. Kidney Blood Press Res. 2001; 34:69-74.

124. Dong GH, Sun ZQ, Zheng LQ, Li Y, Zhang XZ, et al. Prevalence, awareness, treatment, and control of hypertension in rural adults from Liaoning province. Northeast China. 2007; 30(10): 951-958.
